# TaWRKY13-A Serves as a Mediator of Jasmonic Acid-Related Leaf Senescence by Modulating Jasmonic Acid Biosynthesis

**DOI:** 10.3389/fpls.2021.717233

**Published:** 2021-09-01

**Authors:** Hualiang Qiao, Yongwei Liu, Lingling Cheng, Xuelin Gu, Pengcheng Yin, Ke Li, Shuo Zhou, Geng Wang, Chunjiang Zhou

**Affiliations:** ^1^Ministry of Education Key Laboratory of Molecular and Cell Biology, Hebei Innovation Center for Cell Signaling, College of Life Sciences, Hebei Normal University, Shijiazhuang, China; ^2^Institute of Genetics and Physiology, Hebei Academy of Agriculture and Forestry Sciences/Plant Genetic Engineering Center of Hebei Province, Shijiazhuang, China

**Keywords:** wheat, leaf senescence, jasmonic acid, WRKYs, transcriptional regulation

## Abstract

Leaf senescence is crucial for crop yield and quality. Transcriptional regulation is a key step for integrating various senescence-related signals into the nucleus. However, few regulators of senescence implicating transcriptional events have been functionally characterized in wheat. Based on our RNA-seq data, we identified a WRKY transcription factor, TaWRKY13-A, that predominately expresses at senescent stages. By using the virus-induced gene silencing (VIGS) method, we manifested impaired transcription of *TaWRKY13-A* leading to a delayed leaf senescence phenotype in wheat. Moreover, the overexpression (*OE*) of *TaWRKY13-A* accelerated the onset of leaf senescence under both natural growth condition and darkness in *Brachypodium distachyon* and *Arabidopsis thaliana*. Furthermore, by physiological and molecular investigations, we verified that TaWRKY13-A participates in the regulation of leaf senescence *via* jasmonic acid (JA) pathway. The expression of JA biosynthetic genes, including *AtLOX6*, was altered in *TaWRKY13-A*-overexpressing *Arabidopsis*. We also demonstrated that TaWRKY13-A can interact with the promoter of *AtLOX6* and *TaLOX6* by using the electrophoretic mobility shift assay (EMSA) and luciferase reporter system. Consistently, we detected a higher JA level in *TaWRKY13-A*-overexpressing lines than that in Col-0. Moreover, our data suggested that TaWRKY13-A is partially functional conserved with AtWRKY53 in age-dependent leaf senescence. Collectively, this study manifests TaWRKY13-A as a positive regulator of JA-related leaf senescence, which could be a new clue for molecular breeding in wheat.

## Introduction

Leaf senescence is a highly regulated developmental process and triggered by diverse environmental factors (Woo et al., 2019). As a sophisticated biological event, leaf senescence comprises multidimensional alterations in cell structure, metabolism, and expression of genes (Mayta et al., 2019). Organelles and macromolecular substances gradually degraded in an ordered manner. Then, nutrients transfer from senescent parts to developing and storage organs (Thomas and Ougham, 2014; Kim et al., 2018; Koyama, 2018; Zhang et al., 2018b; Woo et al., 2019). Meanwhile, the initiation and progression of leaf senescence are also governed by numerous senescence-related genes that function in phytohormone pathways, transcriptional regulation, epigenetic modification, autophagy, circadian clock, DNA damage repair, and chlorophyll metabolism and light (Jia et al., 2019; Li et al., 2020; Xie et al., 2020; Xu et al., 2020; Yuan et al., 2020).

To date, more and more mechanistic details about how phytohormones regulate leaf senescence have been clarified. Phytohormones directly or indirectly regulate the onset and progression of leaf senescence by fine-tuning developmental programs or responses to stress (Jibran et al., 2013; Smith et al., 2017; Zhang et al., 2020). Different hormones participate in the regulation of leaf senescence with a distinct mechanism. Hormones, such as ethylene, abscisic acid, jasmonic acid (JA), salicylic acid (SA), brassinosteroids, and strigolactones, promote the initiation of leaf senescence, whereas cytokinins (CKs), gibberellins, and auxins delay leaf senescence (Arrom and Munne-Bosch, 2012; Khan et al., 2014; Tan et al., 2019; Wojciechowska et al., 2020; Zhang et al., 2021).

Jasmonic acid as a lipid-derived hormone has been long known for its crucial role in plant development and stress responses. JA is generally considered to be synthesized from alpha-linolenic acid that is further catalyzed by 13-lipoxygenase (LOX), allene oxide synthase, and allene oxide cyclase and then converted to (9S,13S)-12-oxo-phytodienoic acid (OPDA). After undergoing a series of reduction and oxidation reactions, OPDA is changed into JA in peroxisomes. Then, JA is transferred to the cytoplasm and conjugated with isoleucine to form (+)-7-*iso*-JA-Ile (JA-Ile). Meanwhile, JA-Ile can be inactivated by CYP94B3 (Khan et al., 2014; Wasternack and Song, 2016; Huang et al., 2017). To date, many studies have demonstrated that the JA pathway is involved in the regulation of leaf senescence (He et al., 2002b; Fonseca et al., 2009; Wasternack and Hause, 2013; Ahmad et al., 2016; Chini et al., 2016; Wojciechowska et al., 2018; Ruan et al., 2019; Aubry et al., 2020). For instance, some genes related to JA biosynthesis are upregulated during leaf senescence to varying degrees, such as *AtLOX1, AtLOX2, AtLOX3, AtLOX4*, and *AtAOC1* (He et al., 2002b; Kim et al., 2015; Hu et al., 2017). Moreover, TCP4 affects JA biosynthesis by interacting with *LOX2* and thus participates in the regulation of leaf senescence in *Arabidopsis* (Schommer et al., 2008; Koyama et al., 2017). Meanwhile, as positive regulators of the JA signaling pathway, MYC2, MYC3, and MYC4 can directly regulate the expression of *senescence-associated genes* (*SAG*s) by binding their G-box/G-box-like motifs (Qi et al., 2015; Liu et al., 2016; Song et al., 2017; Uji et al., 2017). However, leaf senescence is nearly unaffected by missing some key components of JA signaling transduction and biosynthesis (He et al., 2002a; Seltmann et al., 2010). Thus, more details about JA-related leaf senescence need to be carefully inspected and discussed. Importantly, although the roles of some genes in integrating the JA pathway with leaf senescence have been functionally studied in *Arabidopsis*, the mechanism underlying JA-related leaf senescence in wheat is still obscure.

WRKY transcription factors (TFs) are one of the largest TFs in plants, which play vital roles in many biological processes, including leaf senescence (Lin and Wu, 2004; Li et al., 2018). WRKY TFs contain the WRKY domain (a conserved amino acid sequence of WRKYGQK) at the N-terminus and an atypical zinc finger domain at the C-terminus. WRKY proteins are initially divided into three groups as follows: the first group contains a C_2_H_2_ (CX_4−5_CX_22−23_HX_1_H) zinc finger motif and a WRKY domain, the second group contains a C_2_H_2_ zinc finger motif and two WRKY domains, and the third group contains a C_2_-HC (CX_7_Cx_23_HX_1_C) zinc finger motif. Recently, the phylogenetic analysis among different plant species suggested that WRKY protein should be divided into groups I, IIa + IIb, IIc, IId + IIe, and III. WRKYs generally bind to the W-box (TTGACC/T) in diverse target genes and hence mediate various signals (Eulgem et al., 2000; Rushton et al., 2010; Jiang et al., 2017; Song et al., 2018). To date, the functional role of some WRKYs in the regulation of leaf senescence has been predominately demonstrated in *Arabidopsis* (Hinderhofer and Zentgraf, 2001; Schippers, 2015). Among the senescence-related WRKYs, AtWRKY53 functions as a central regulator and integrates many senescence-related signals at the transcriptional level (Miao and Zentgraf, 2007; Zheng et al., 2020). AtWRKY45, AtWRKY57, and AtWRKY75 regulate the initiation of leaf senescence *via* phytohormone pathways (Jiang et al., 2014; Chen et al., 2017; Guo et al., 2017). AtWRKY54 and AtWRKY70 cooperatively suppress the onset of leaf senescence (Besseau et al., 2012). AtWRKY6 promotes leaf senescence but it is repressed by DELLA proteins (Robatzek and Somssich, 2001; Lim et al., 2018; Zhang et al., 2018c). AtWRKY55 positively regulates leaf senescence by affecting reactive oxygen species and SA level (Wang et al., 2020). Although many senescence-related WRKYs have been functionally characterized in *Arabidopsis*, WRKYs implicated in the regulation of leaf senescence are extremely elusive in wheat.

Common wheat (*Triticum aestivum* L.) is one of the most widely cultivated food crops. However, due to the allohexaploid genome of wheat, studies on candidate genes of various biological processes are difficult to carry out. Hence, some functional studies on wheat genes are also conducted with the help of some analysis in other monocots. For instance, as a model plant of monocot grass, *Brachypodium distachyon* possesses a much smaller genome than wheat and is more easily to be transformed (Scholthof et al., 2018). Thus, the experimental data from *B. distachyon* are also significantly helpful to understand the mechanistic framework of leaf senescence in wheat. To date, as more and more detailed information on the wheat genome is available, researchers have identified some key components of different regulatory networks in wheat (Borrill et al., 2019; Sultana et al., 2021). *NAM-B1* is reported to accelerate leaf senescence onset and promote nutrients redistribution (Uauy et al., 2006). The wheat copper-binding protein (WCBP1) is tightly related to the regulation of leaf senescence when wheat plants undergo the infection of stripe rust (Li et al., 2015). Our data reveal TaWRKY42-B and TaWRKY40-D as positive regulators in phytohormone-related wheat leaf senescence (Zhao et al., 2020a,b). Moreover, cisZOGT1, a *cis*-zeatin *O*-glucosyltransferase, is involved in wheat leaf senescence by regulating CK and N metabolism (Wang et al., 2019). By the high-throughput analysis, researchers have also identified some candidate genes in drought-induced leaf senescence in wheat (Luo et al., 2019). Meanwhile, TaSCL14 is a member of the GRAS protein family in wheat and plays multiple roles in development, photosynthesis, stress response, and dark-induced senescence (Chen et al., 2015).

In this study, we identified a WRKY type TF, *TaWRKY13-A*, as a positive regulator of leaf senescence under both natural condition and darkness. *TaWRKY13-A*-silenced wheat plants showed the delayed leaf senescence phenotype. Consistently, the overexpression of *TaWRKY13-A* promoted leaf senescence in *B. distachyon* and *Arabidopsis*. Furthermore, we manifested that *TaWRKY13-A* regulates leaf senescence by targeting JA biosynthetic genes. By affecting the expression of *LOXs*, TaWRKY13-A can enhance the JA content, which finally contributes to the initiation and progression of leaf senescence. Our data also suggested that TaWRKY13-A is partially conserved with AtWRKY53.

## Materials and Methods

### Plant Materials and Growth Conditions

The *Arabidopsis* seeds were sterilized with ethanol and sprinkled on 1/2MS solid medium. The above seeds were placed at 4°C under darkness for 2 days and then continued to grow in a growth chamber for the next 5 days. Seven-day-old seedlings were transferred to a greenhouse at 22°C (16-h light/8-h dark) for the subsequent cultivation. *Arabidopsis thaliana* Col-0 and *atwrky53* (SALK_034157) seeds used in this study were obtained from Arabidopsis Biological Resource Center and provided by Prof. Ying Miao (Fujian Agriculture and Forestry University). The background of *atwrky53* mutants was confirmed with PCR assay by following the published data (Miao and Zentgraf, 2007).

Bread wheat seeds germinated and were grown to the two-leaf stage in water, and then they were transferred into the greenhouse at 25°C, with the humidity at 70% and in the period of 16/8 h light/dark. Bread wheat varieties “ShiLuan 02-1” were provided by Prof. Zhanjing Huang (Hebei Normal University), and “cv. Chinese spring,” “KeNong199” was preserved and obtained from the seed bank of the Institute of Genetics and Physiology, Hebei Academy of Agriculture and Forestry Sciences. Wheat plants of “ShiLuan02-1” were used for the expression mode analysis of *TaWRKY13-A*. The 10-day-old etiolated seedlings of “KeNong199” were used to generate wheat protoplasts.

### Plasmid Construction and Plant Transformation

The full-length coding sequences (CDS) of *TaWRKY13-A* was constructed into the pCAMBIA1300-MYC-HIS vector and driven by using the 35S promoter (Supplementary Figure 3C). Vectors were transformed into Col-0 and *atwrky53* mutants by *Agrobacterium* stain GV3101 by using the floral dip transformation method (Clough and Bent, 1998).

For the transcription activation assay, the CDS of *TaWRKY13-A* was fused with the GAL4 DNA binding domain in pSAT-GAL4DB.

For the subcellular localization analysis, the full-length *TaWRKY13-A* CDS was constructed into the pUC19 vector and transformed into wheat protoplasts to observe the subcellular localization of TaWRKY13-A.

To express and purify the MBP-TaWRKY13-A fusion protein for the electrophoretic mobility shift assay (EMSA), the open reading frame sequence of *TaWRKY13-A* was cloned into the pMAL-C2X expression vector and transformed into *Escherichia coli* (strain *Rosetta*) competent cells for the prokaryotic expression.

### Ion Leakage and Chlorophyll Content

The chlorophyll content was measured by using a chlorophyll meter (SPAD 502 Plus Chlorophyll Meter, Minolta Corporation, Tokyo, Japan). First, leaves were placed in 10 ml of deionized water and vacuumed for 1 h, and then the conductivity was measured. Then, leaves were boiled for 10 min and the conductivity was measured again after the water cooled down. Ion leakage rate was indicated by the ratio of conductivity of leaves before boiled/after boiled in deionized water.

### Quantitative Real-Time-PCR

By using Trizol (Takara, 9109), the total RNA was extracted from *Arabidopsis*, wheat, and *Brachypodium*. A total of 500 ng of RNA was used to generate cDNA by using 5 × HiScriptII qRT SuperMixII (R223-01). The real-time PCR analysis was performed on a CFX96 real-time fluorescent quantitative PCR instrument by using 2 × ChamQ Universal STBR Master Mix (Q711-02/0).

All the primers used in this study are listed in Supplementary Table 1. In the quantitative real-time (qRT)-PCR analysis, each sample was tested in three technical and three biological repeats. In wheat, the expression of the *TaACTIN* gene is used as an internal control, while in *Arabidopsis*, it is *AtUBC30*.

### Quantification of JA Content

To analyze the JA content in *TaWRKY13-A*-*OE* and Col-0 *Arabidopsis* plants, fifth and sixth leaves of 4-week-old and 5-week-old *Arabidopsis* were selected for liquid chromatography-tandem mass spectrometry (LC-MS/MS) assay. A total amount of 200 mg leaves of the above plants were grounded and incubated with methanol for 24 h. By using the Oasis Max solid-phase extraction cartridge, all samples were purified. The JA content was measured using the ultra-performance liquid chromatography (UPLC) system (Waters) (Agilent Technologies Inc, California, USA) and QTRAP 6500 system (AB SCIEX, Framingham, MA, USA). The measurement of each sample was repeated in three biological replicates, and ^2^H_5_-JA was used as the internal reference. The JA content of each sample was finally examined by ultra-performance liquid chromatography-mass spectrometry/mass spectrometry (UPLC-MS/MS) (Waters) and QTRAP 6500 system (AB SCIEX).

### Barley Stripe Mosaic Virus-Virus-Induced Gene Silencing

The vectors for Barley stripe mosaic virus (BSMV)–virus-induced gene silencing (VIGS) were provided by Prof. Dawei Li. A 326 bp fragment amplified from TaWRKY13-A cDNA was introduced into the pCaBS-γbLIC vector. *Agrobacterium* containing each pCaBS-α, β, and γ vector was cultured on a shaker overnight and collected. Each of the above bacterial solutions [10 mM MES, 10 mM MgCl_2_, pH 5.2, and 0.1 mM Acetosyringone (AS)] was adjusted to Optical Density (OD) = 0.7, mixed with the others, and incubated at 30°C for 5 h. The mixed solution was further injected into 2-week-old tobacco leaves. Two weeks later, we grounded the infected tobacco leaves with PBS buffer and then injected it into the two-leaf wheat seedlings. The wheat plants harboring the empty vectors (i.e., pCaBS-α, pCaBS-β, and pCaBS-γbLIC) were used as negative controls.

### Electrophoretic Mobility Shift Assay

The protein used in the EMSA experiment was purified using the Amylose Resin (0812S, New England Biolabs, Beverly, MA, USA). The CDS of *TaWRKY13-A* was subcloned into the pMal-c2X vector and transformed into *Rosetta* strain. After the addition of Isopropyl-beta-D-thiogalactopyranoside (IPTG) (final concentration of 1 mM), MBP-TaWRKY13-A was expressed at 18°C for 5 h and purified. The probes used in the EMSA experiment (Supplementary Table 1) were all labeled with biotin at the 5′end. We performed the EMSA by using the Chemiluminescent Nucleic Acid Detection Module (Thermo Scientific, 89,880) to detect the interaction between protein and DNA. The total reaction system is 10 μl, including 1 μl of binding buffer, 0.5 μl of poly-dIdC, 0.5 μl of glycerol, 0.5 μl of 1 M KCl, 1 μl of biotin-probe, and 400 ng of the target protein. Unlabeled probes were added at 100- and 200-fold of labeled probes as competitors. The mixture was placed at 4°C for 20 min and subjected to the electrophoresis analysis. Biotin-labeled probes are listed in Supplementary Table 1.

## Results

### Identification and Sequence Analysis of *TaWRKY13-A*

To identify WRKY TFs related to leaf senescence in wheat, we analyzed our RNA-seq data at four developmental stages of flag leaves (i.e., YL, young leaves with half size of mature leaves; ML, mature leaves, fully expanded leaves; ES, early senescence leaves with <25% leaf area yellowing; and LS, late senescence leaves with >50% leaf area yellowing) in wheat (cv. Chinese Spring) (Zhao et al., 2020b) and selected a WRKY TF (TraesCS4A02G193600.1) that shows a more significantly increasing expression trend during leaf senescence than its paralogs on chromosomes B and D (Supplementary Figures 1B,C). Hence, in this study, we mainly focused on this gene. According to the corresponding sequence obtained from the WheatOmics (http://202.194.139.32/), we confirmed that the candidate gene is *TaWRKY13-A* that encodes a 24.49-kDa protein of 222 amino acids and with an isoelectric point of 8.33. The presence of a WRKY domain and a C_2_HC zinc finger motif indicated that TaWRKY13-A is a member of the group III WRKYs (Supplementary Figure 3A). Then, we performed a phylogenetic analysis among the amino sequence of TaWRKY13-A and some published senescence-related WRKYs, and we found that TaWRKY13-A is relatively close to AtWRKY55, AtWRKY70, AtWRKY54, and AtWRKY53 (Supplementary Figure 1A).

### Spatiotemporal Expression Pattern of TaWRKY13-A

**Figure 1:**
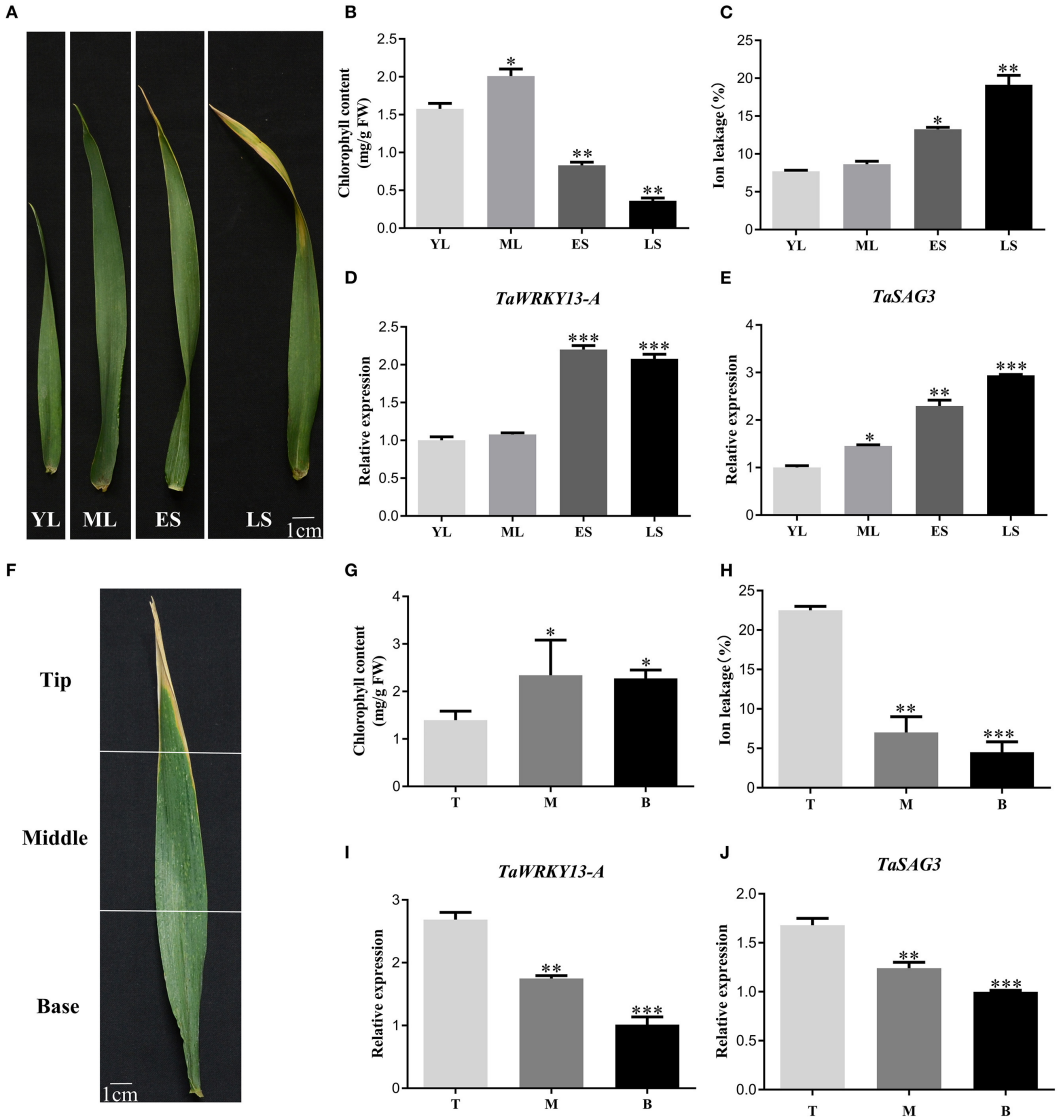
Expression pattern of *TaWRKY13-A* in wheat. **(A)** Four different development stages of flag leaf (i.e., YL, young leaves with half size of mature leaves; ML, mature leaves, fully expanded leaves; ES, early senescence leaves with <25% leaf area yellowing; and LS, late senescence leaves with >50% leaf area yellowing) in cv. Chinese spring. **(B,C)** Chlorophyll content and ion leakage rate of **(A)**. **(D)** Transcription level detection of *TaWRKY13-A* in **(A)** by using the quantitative real-time (qRT)-PCR. **(E)** Transcription level detection of a *senescence-associated gene, TaSAG3*, in **(A)** by using the qRT-PCR. **(F)** The tip, middle, and base of a senescent wheat flag leaf. **(G,H)** Chlorophyll content and ion leakage rate of **(F)**. **(I)** Transcription level detection of *TaWRKY13-A* expression in **(F)** by qRT-PCR. **(J)** Transcription level detection of *TaSAG3* in **(F)** by using the qRT-PCR. (Error bars indicate SD. Asterisks indicate significant differences. Student's *t*-test, ^*^P < 0.05, ^**^P < 0.01, ^***^P < 0.001, and *n* ≥ 30. Above experiments were repeated at least in three biological replicates).

**Figure 2:**
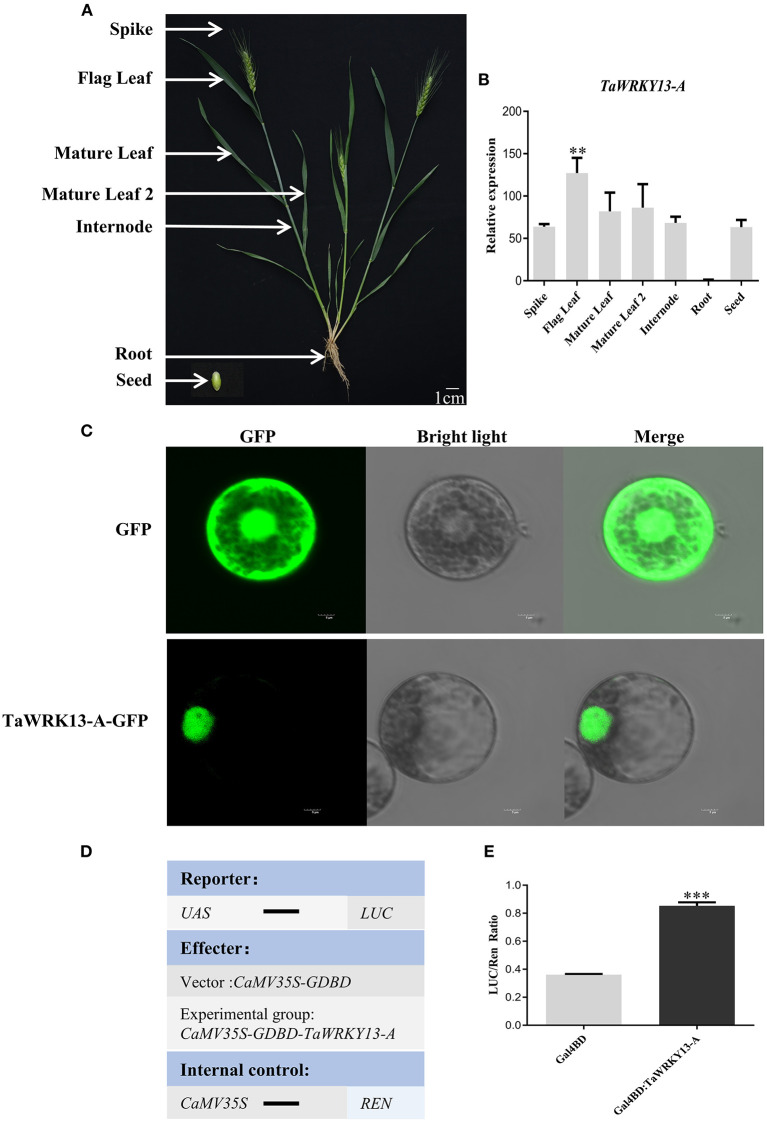
Expression pattern and subcellular localization analysis of TaWRKY13-A. **(A)** Different tissues of 6-month-old mature wheat. **(B)** The tissue expression pattern of *TaWRKY13-A* in **(A)** by using the qRT-PCR (Flag leaf, Spike, 1st mature leaf from the top, Mature leaf 1; 2nd mature leaf from the top, Mature leaf 2; stem, root, and seed). **(C)** Subcellular localization of TaWRKY13-A in wheat protoplasts (scale bar = 5 μm). **(D,E)** Transcriptional activity assay of TaWRKY13-A in wheat protoplasts. Schematic diagrams indicate the vector constructs. Measurement of relative activity LUC after transient expression of fusion vector in wheat protoplasts. (Error bars indicate SD. Asterisks indicate significant differences. Student's *t*-test, ^**^P < 0.01, ^***^P < 0.001, and *n* ≥ 30. The above experiments were repeated at least in three biological replicates).

To investigate the role of TaWRKY13-A, we first checked the expression profiling of *TaWRKY13-A* in wheat flag leaves at four different developmental stages (i.e., YL, ML, ES, and LS) (Figure 1A). Parameters related to leaf senescence, including chlorophyll content, ion leakage rate (Figures 1B,C), and the transcription level of a senescence marker gene *TaSAG3*, were measured to verify the accuracy of harvesting different leaves (Figure 1E). Consistent with the information on WheatOmics (http://202.194.139.32/) and our RNA-seq data (Supplementary Figures 1B,C), we confirmed that *TaWRKY13-A* predominantly expressed at ES and LS stages by the qRT-PCR assay (Figure 1D). In general, the onset of senescence is from the tip of a leaf and gradually proceeds to the leaf base (Figure 1F). Consistently, we measured the most chlorophyll content and the least ion leakage rate in the leaf tip (Figures 1G,H). We also detected more *TaWRKY13-A* transcripts in the leaf tip than in the middle and base (Figure 1I), which is in line with the expression of *TaSAG3* (Figure 1J). Then, we analyzed the transcription level of *TaWRKY13-A* in different tissues, including spike, seed, root, internode, flag leaf, and mature leaf (Figure 2A). We detected a ubiquitous expression pattern of *TaWRKY13-A*, while transcripts of *TaWRKY13-A* were predominantly concentrated in flag leaves (Figure 2B). The above results indicated that *TaWRKY13-A* may play a role in wheat leaf senescence.

### TaWRKY13-A Localizes in the Nucleus and Possesses Transcriptional Activity

It is widely acknowledged that WRKYs are responsible for mediating diverse signals at the transcriptional level. To investigate whether TaWRKY13-A has the potential to regulate transcriptional events, we generated a *35S:TaWRK13-A-GFP* construct, which was transformed and transiently expressed in wheat protoplasts. Fluorescent signals of TaWRKY13-A-GFP fusion appeared only in the nucleus, while the single GFP protein was detectable among plasma membrane, cytoplasm, and nucleus (Figure 2C). To further verify whether TaWRKY13-A functions as a TF, we used the dual-luciferase reporter system to test the transcriptional activity of TaWRKY13-A in wheat protoplasts. We fused the TaWRKY13-A with Gal4-DNA binding domain (GDBD) and then transformed GDBD-TaWRKY13-A with the *firefly luciferase* (*LUC*) gene driven by a fusion of CaMV 35S promoter and upstream activation sequence. The 35S:*Renilla luciferase* (*REN*) construct served as an internal control (Figure 2D). We found that the activity ratio of LUC/REN was specifically elevated by GDBD-TaWRKY13-A (Figure 2E). These results suggested that TaWRKY13-A may function as a TF.

### Silencing of *TaWRKY13-A* Causes the Delayed Leaf Senescence Phenotype in Wheat

**Figure 3:**
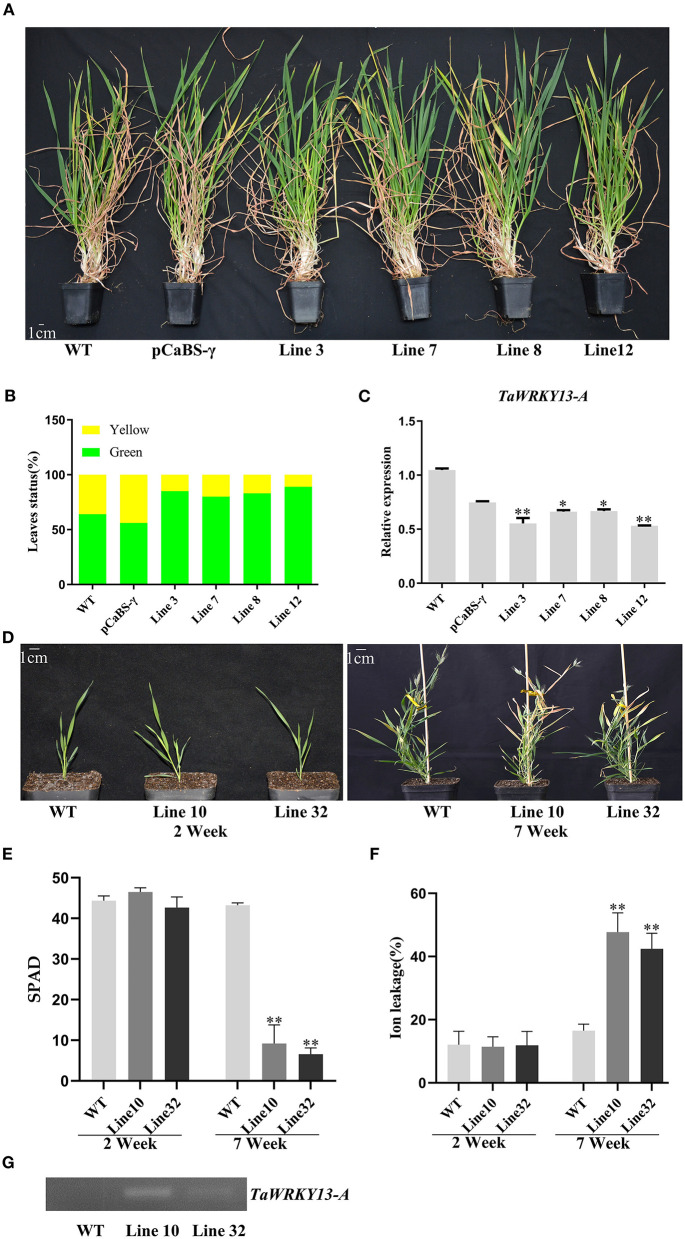
Phenotypic observation in *TaWRKY13-A*-silenced wheat and *TaWRKY13-A*-overexpressing *Brachypodium distachyon*. **(A)** Observation of the leaf senescence phenotype in *TaWRKY13*-A-silenced (VIGS technology) wheat leaves under natural conditions. **(B)** Statistics of green leaves and senescent leaves (yellow of 9-week-old wheat plants in **(A)**. **(C)** Expression of *TaWRKY13-A* in *TaWRKY13-A*-silenced wheat by using the qRT-PCR. **(D)** Observation of the leaf senescence phenotype in *TaWRKY13-A*-overexpressing *B. distachyon*. **(E,F)** Physiological data measurements in **(D)** including chlorophyll content **(E)** and ion leakage rate **(F)**. **(G)** Detections of the overexpression of *TaWRKY13-A* at transcription levels in *B. distachyon*. (Error bars indicate SD. Asterisks indicate significant differences. Student's *t*-test, ^*^P < 0.05, ^**^P < 0.01, and *n* ≥ 30. The above experiments were repeated at least in three biological replicates).

To further evaluate the function of TaWRKY13-A, we silenced TaWRKY13-A in wheat by using the BSMV–VIGS method. Bleached leaves induced by the impairment of the *TaPDS* gene indicated that the BSMV–VIGS method used in this study is feasible (Supplementary Figures 4A,B). Hence, we selected a 326 bp target sequence from 342 bp downstream of the translation initiation codon of TaWRKY13-A for BSMV–VIGS. However, due to the high similarity between *TaWRKY13-A* and *TaWRKY13-B*, we were not able to select a unique target sequence only in *TaWRKY13-A* cDNA. One-week-old wild-type (WT) wheat plants and wheat seedlings that were infected by BSMV that contains *TaWRKY13-A*_326_ (pCaBS-α, pCaBS-β, and pCaBS-γbTaWRKY13-A_326_) or empty vector (pCaBS-γbLIC) were used in this research. By using the qRT-PCR assay, we selected all the wheat plants with the decreased transcription level of *TaWRKY13-A* among those infected plants for subsequent analysis. After 25 days of growth, the eighth leaf from the top of *TaWRKY13-A*-silenced plants and control groups were used to compare the dark-induced leaf senescence phenotype. After 6 days under darkness, leaf senescence was remarkably accelerated in *TaWRKY13-A*-silenced leaves when compared with that in vector control (VC) and WT (Supplementary Figure 4C). Meanwhile, chlorophyll degradation and ion leakage rate were in line with the phenotypic changes (Supplementary Figures 4D,E). Moreover, we counted the number of senescent and non-senescent leaves among 9-week-old *TaWRKY13-A*-silenced plants and control groups (Figure 3C). The statistical data showed a significantly lower ratio of yellow/green leaves in *TaWRKY13-A*-silenced plants than that in control groups (Figure 3B). These results suggested that TaWRKY13-A is involved in the regulation of leaf senescence in wheat.

### Overexpression of *TaWRKY13-A* Promotes Age-Dependent and Dark-Induced Leaf Senescence in *B. distachyon* and *Arabidopsis*

To further assess the role of TaWRKY13-A in leaf senescence, we produced *TaWRKY13-A*-overexpressing (*OE*) lines in *B. distachyon* and *Arabidopsis*. First, we generated a construct where the fusion of full-length *TaWRKY13-A* CDS and Flag tag is under the control of the ubiquitin (Ubi) promoter. The constructs of P_Ubi_:TaWRKY13-A-Flag were further transformed into *Brachypodium* callus. The expression of *TaWRKY13-A* was confirmed by semi-quantitative reverse transcript PCR (RT-PCR) (Figure 3G). Two *TaWRKY13-A*-overexpression *Brachypodium* lines (i.e., Line 10 and Line 32) and WT both exhibited normal growth at the seedling stage. While 7 weeks after sowing, Line 10 and Line 32 showed the obviously precocious leaf senescence phenotype when compared with WT (Figure 3D). The chlorophyll content and cell membrane integrity were significantly lower than those in WT at the senescent stage (Figures 3E,F). Dark-induced leaf senescence was also assessed among detached leaves of Line 10, Line 32, and WT. After treatment, leaf senescence triggered by darkness appeared earlier in Line 10 and Line 32 than that in WT (Supplementary Figure 5A). Consistently, chlorophyll degradation and ion leakage were more severe in Line 10 and Line 32 than those in WT (Supplementary Figures 5B,C).

**Figure 4:**
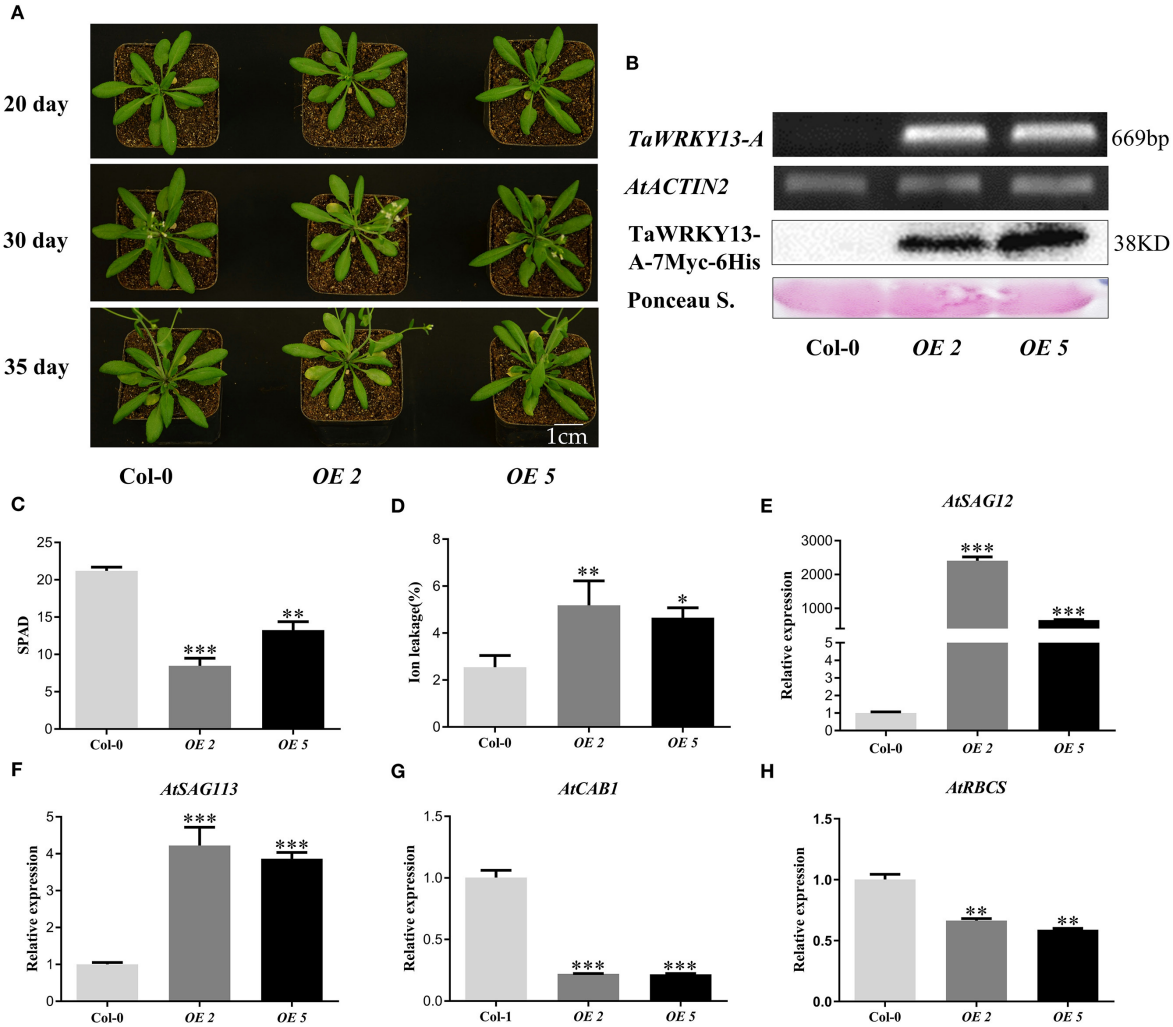
Phenotypic observation in *TaWRKY13-A*-overexpressing *Arabidopsis*. **(A)** Observation of leaf senescence phenotype of *TaWRKY13-A*-overexpressing *Arabidopsis* and Col-0 under the natural growth condition. **(B)** Detections of the overexpression of *TaWRKY13-A* at transcription and protein levels in *Arabidopsis*. **(C,D)** Physiological data measurement in **(A)** including chlorophyll content **(C)** and ion leakage rate **(D)**. **(E,H)** Detection of senescence-associated genes including up-regulated genes *AtSAG12* **(E)**, *AtSAG113* **(F)** and downregulated genes *AtCAB1* **(G)**, *AtRBCS* **(H)**. (Error bars indicate SD. Asterisks indicate significant differences. Student's *t*-test, ^*^P < 0.05, ^**^P < 0.01, ^***^P < 0.001, and *n* ≥ 30. The above experiments were repeated at least in three biological replicates).

Moreover, the full-length 669 bp CDS of *TaWRKY13-A* was cloned into pCAMBIA1300 and fused with the 7Myc-6His tag. This *TaWRKY13-A-7Myc6His* fusion was driven by using the CaMV 35S promoter. Two independent homozygous transgenic *Arabidopsis* lines (*OE-2* and *OE-5*) were selected for phenotypic and physiological analysis. We confirmed the increased expression level of *TaWRKY13-A*-overexpressing lines by using the RT-PCR and Western blot (Figure 4B). Then, we observed that 5-week-old *OE-2* and *OE-5* plants exhibited obviously early leaf senescence when compared with Col-0 (Figure 4A). Consistently, the chlorophyll content in *TaWRKY13-A*-overexpressing lines was lower than that in Col-0 (Figure 4C), and the overexpression of *TaWRKY13-A* also accelerated ion leakage (Figure 4D). Additionally, the expression level of two *SAGs*, namely, *AtSAG12* and *AtSAG113*, in *TaWRKY13-A*-overexpressing lines were higher than those in Col-0 (Figures 4E,F), while two senescence downregulated genes, namely, *AtRBCS* and *AtCAB1*, were decreased in *OE-2* and *OE-5* when compared with those in Col-0 (Figures 4G,H). To investigate whether TaWRKY13-A is also involved in dark-induced leaf senescence in *Arabidopsis*, we covered the fifth and sixth leaves on 4-week-old *OE-2, OE-5*, and Col-0 by using the aluminum foil for 6 days. Meanwhile, we also harvested the fifth and sixth rosette leaves of 4-week-old *TaWRKY13-A*-overexpressing lines and Col-0 for treatment under darkness. Then, these phenotypically indistinguishable leaves were incubated under darkness for 6 days (Supplementary Figures 6A,D). After treatment, leaves of *OE-2* and *OE-5* showed a significantly precious leaf senescence compared with WT leaves. Chlorophyll degradation (Supplementary Figures 6B,E) and ion leakage rate altered more dramatically than those in Col-0 (Supplementary Figures 6C,F). All above data proved that TaWRKY13-A can promote leaf senescence under both natural growth conditions and darkness. Moreover, the functional role of TaWRKY13-A in leaf senescence seemed to be conserved in *B. distachyon* and *Arabidopsis*, which helps us to screen the target genes of TaWRKY13-A in leaf senescence with the help of some more convenient strategies than only in wheat.

### Inducible Overexpression of *TaWRKY13-A* Promotes Leaf Senescence

To rule out the effect of constitutive expression of *TaWRKY13-A* by CaMV 35S promoter, we generated two inducible *TaWRKY13*-A-overexpressing lines (i.e., *iOE-1* and *iOE-5*). The CDS of *TaWRKY13-A* was under the control of the dexamethasone (DEX)-inducible promoter, thus the expression of *TaWRKY13-A* was rapidly induced by exogenous application of 30 μM DEX (Supplementary Figure 7E). Transgenic plants harboring empty vectors were used as VC. We sprayed dexamethasone on 28-day-old *iOE-1, iOE-5*, VC, and Col-0 *Arabidopsis* plants. Compared with VC and Col-0, *iOE-1* and *iOE-5* showed the significantly premature phenotype at 4 days after the application of DEX (Supplementary Figure 7A). We detected the significantly reduced chlorophyll level (Supplementary Figure 7C) and the higher membrane ion leakage rate (Supplementary Figure 7D) in *iOE-1* and *iOE-5* when compared with control groups. Generally, the H_2_O_2_ level is increasing with the progression of leaf senescence. Thus, we performed the 3,3-diaminobenzidine staining among *iOE-1, iOE-5*, VC, and Col-0 to indicate the H_2_O_2_ level *in vivo*. Compared with VC and Col-0, more dark brown spots were detected in *iOE-1* and *iOE-5* (Supplementary Figure 7B). Furthermore, the expression level of senescence-specific marker genes including *AtSAG12, AtSAG13, AtSAG113, AtCAB1*, and *AtRBCS* in leaves of *iOE-1, iOE-5*, VC, and Col-0 was analyzed. We confirmed that *AtSAG12, AtSAG13*, and *AtSAG113* in *iOE-1* and *iOE-5* were enhanced compared with VC and Col-0 (Supplementary Figures 7F–H). Transcriptions of two senescence downregulated genes, namely, *AtCAB1* and *AtRBCS*, were strongly reduced by overexpression of *TaWRKY13-A* (Supplementary Figures 7I,J). The above results further illustrated that TaWRKY13-A can specifically function in leaf senescence.

### TaWRKY13-A Promotes Leaf Senescence by Upregulating JA Pathway Genes

**Figure 5:**
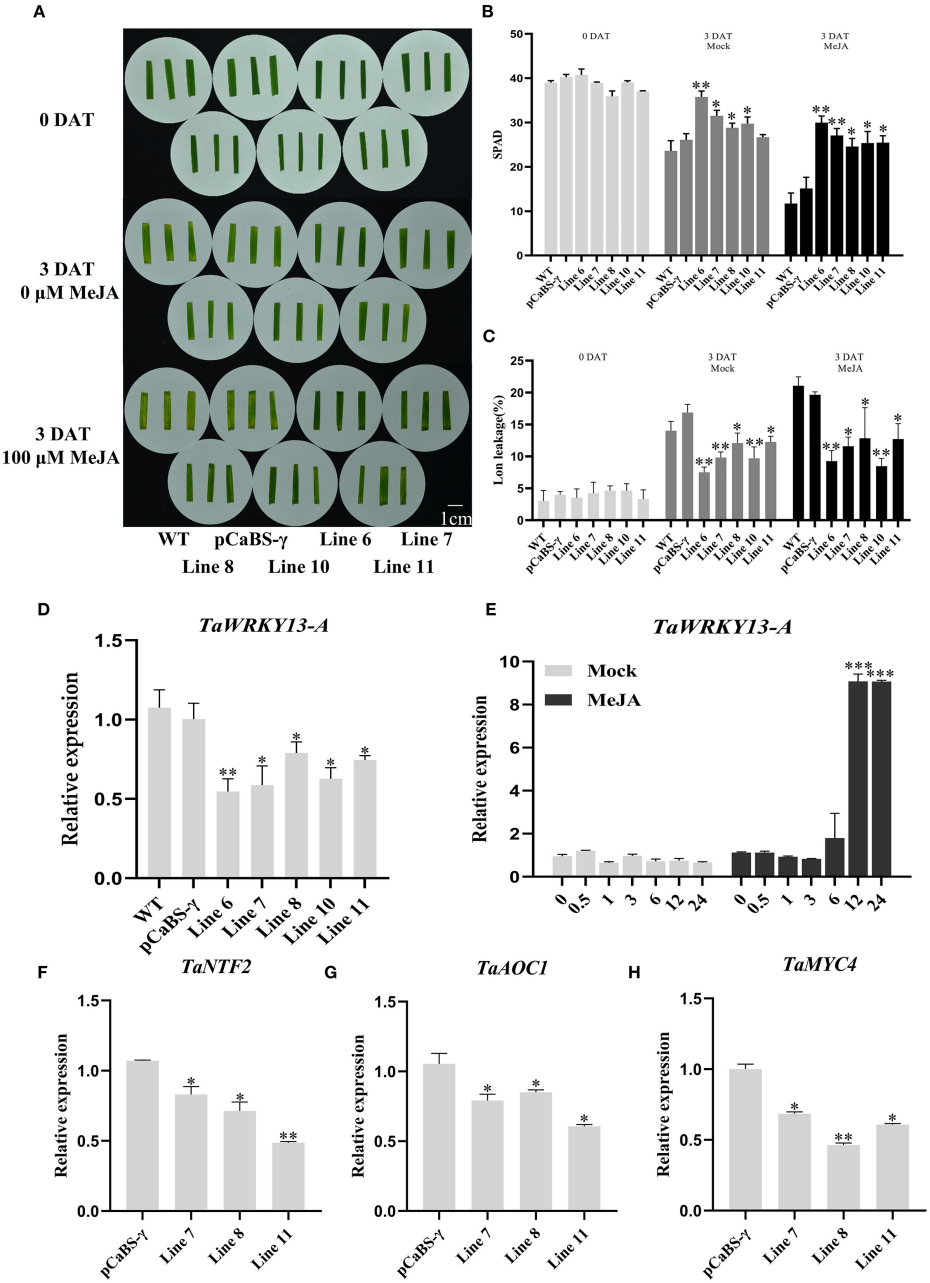
*TaWRKY13-A*-silenced wheat is insensitive to MeJA treatment. **(A)** Observation of the leaf senescence phenotype of *TaWRKY13-A*-silenced wheat and control groups under 100 μM of MeJA treatment. **(B,C)** Physiological data measurement in **(A)** including chlorophyll content **(B)** and ion leakage rate **(C)**. **(D)** Detection of *TaWRKY13-A* transcription in **(A)**. **(E)** Expression pattern of *TaWRKY13-A* under MeJA treatment. **(F–H)** Expression of the JA-responsive gene, *TaNTF2* **(F)**, *TaAOC1* **(G)**, and *TaMYC4* **(H)** in *TaWRKY13*-*A*-silenced and VC wheat plants. (Error bars indicate SD. Asterisks indicate significant differences. Student's *t*-test, ^*^P < 0.05, ^**^P < 0.01, ^***^P < 0.001, and *n* ≥ 30. The above experiments were repeated at least in three biological replicates).

To reveal the mechanism underlying TaWRKY13-A-promoted leaf senescence, we inspected the *cis*-acting elements in the *TaWRKY13-A* promoter region for clues. Notably, eight CGTCA motifs related to JA responsiveness are located at −990 bp, −972 bp, −853 bp, −766 bp, −754 bp, −734 bp, −550 bp, −502 bp, and −454 bp of the TaWRKY13-A promoter (Supplementary Figure 3B). Therefore, we speculated that TaWRKY13-A regulates leaf senescence *via* the JA pathway. First, we examined the expression pattern of *TaWRKY13-A* under 100 μM of MeJA. Transcripts of *TaWRKY13-A* increased from 6 h and reached the peak at 12 h after treatment when compared with the mock (Figure 5E). Then, we analyzed the JA-induced leaf senescence among *TaWRKY13-A*-silenced wheat plants and controls. Leaf senescence was remarkably induced in all plants, while the premature phenotype was most accelerated in controls than *TaWRKY13-A*-silenced wheat (Figures 5A–D). Meanwhile, we examined the expression of three JA-responsive genes, including *TaNTF2, TaAOC1*, and *TaMYC4* between VC and *TaWRKY13-A*-silenced wheat plants (Zhao et al., 2014; Zhang et al., 2018a; Jing et al., 2019). By using the qRT-PCR assay, we detected the significantly lower levels of *TaNTF2, TaAOC1*, and *TaMYC4* in *TaWRKY13-A*-silenced wheat plants when compared with VC plants (Figures 5F–H).

**Figure 6:**
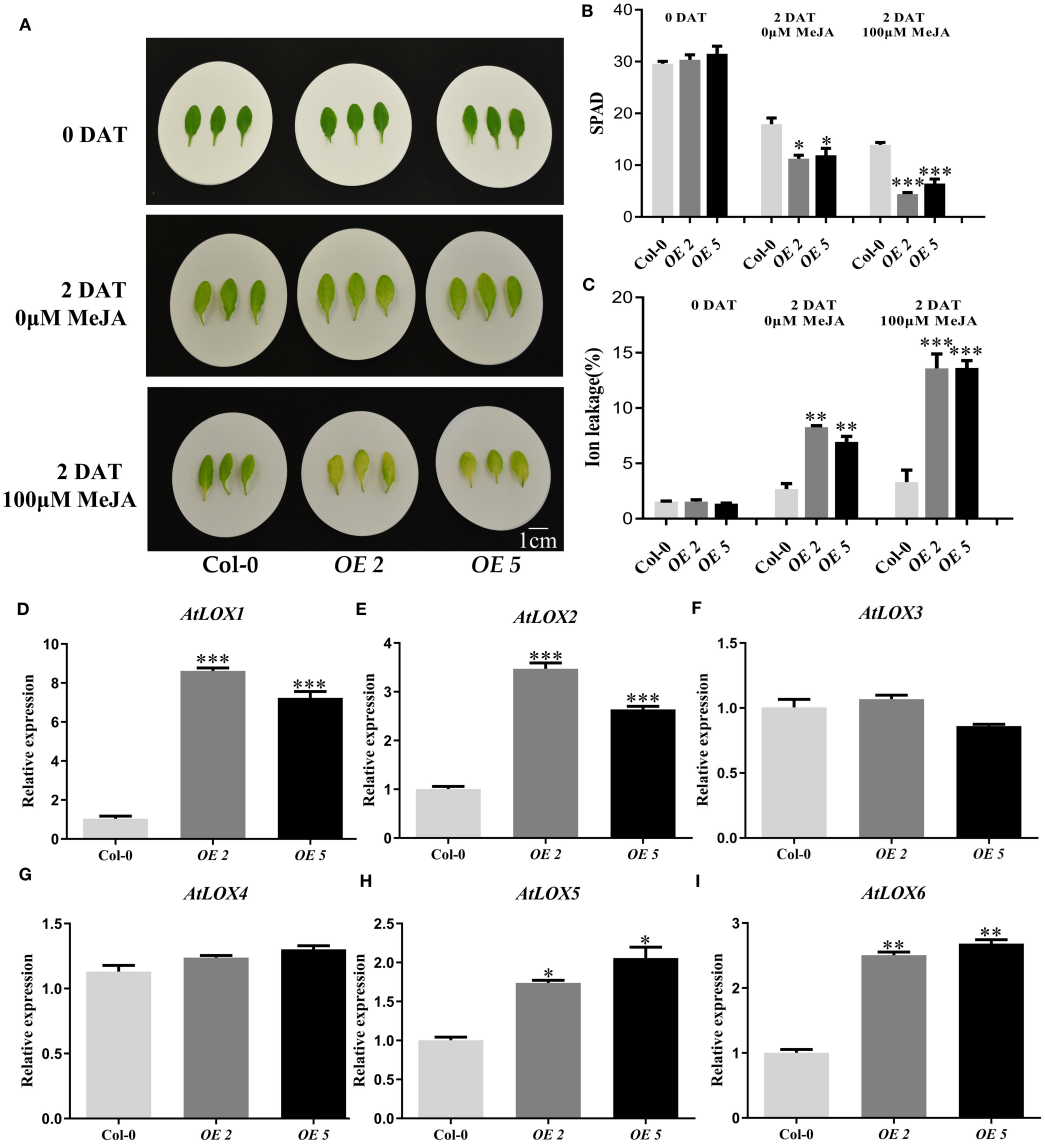
TaWRKY13-A participates in JA-induced leaf senescence in *Arabidopsis*. **(A)** Phenotypic observation of 4-week-old *TaWRKY13-A*-overexpressing *Arabidopsis* and Col-0 under MeJA treatment. **(B,C)** Chlorophyll content **(B)** and ion leakage rate **(C)** before and after MeJA treatment **(A)**. **(D–I)** The transcription level of some JA biosynthetic genes, including *AtLOX1, AtLOX2, AtLOX3, AtLOX4, AtLOX5*, and *AtLOX6*, in the fourth and fifth rosette leaves of 4-week-old T*aWRKY13-A*-overexpressing and Col-0 *Arabidopsis* by qRT-PCR. (Error bars indicate SD. Asterisks indicate significant differences. Student's *t*-test, ^*^P < 0.05, ^**^P < 0.01, ^***^P < 0.001, and *n* ≥ 30. The above experiments were repeated at least in three biological replicates).

In addition, we treated the non-senescent fifth or sixth rosette leaves of 4-week-old *TaWRKY13-A*-overexpressing *Arabidopsis* lines and Col-0 with 100 μM of MeJA for 2 days under darkness (Figure 6A). Leaf senescence was also accelerated by MeJA treatment in all leaves, but chlorophyll degradation and ion leakage in *TaWRKY13-A*-overexpressing plants were more severe than control plants after MeJA treatment (Figures 6B,C).

To further analyze the interaction between the TaWRKY13-A and JA pathways, we detected the expression levels of different genes related to JA signaling transduction and biosynthesis in *TaWRKY13-A*-overexpressing *Arabidopsis* and Col-0. JA biosynthetic genes, including *AtLOX1, AtLOX2, AtLOX5*, and *AtLOX6* in *OE-2* and *OE-5* were enhanced when compared with those in Col-0 (Figures 6D–I). Moreover, signaling components, such as *AtMYC2, AtMYC3, AtMYC4, AtVSP1*, and *AtVSP2*, were also affected by *TaWRKY13-A* overexpression (Supplementary Figures 10A–F). These results indicated that TaWRKY13-A promotes leaf senescence tightly related to the JA pathway.

### TaWRKY13-A Promotes JA Biosynthesis by Binding to Promoters of *LOXs*

**Figure 7:**
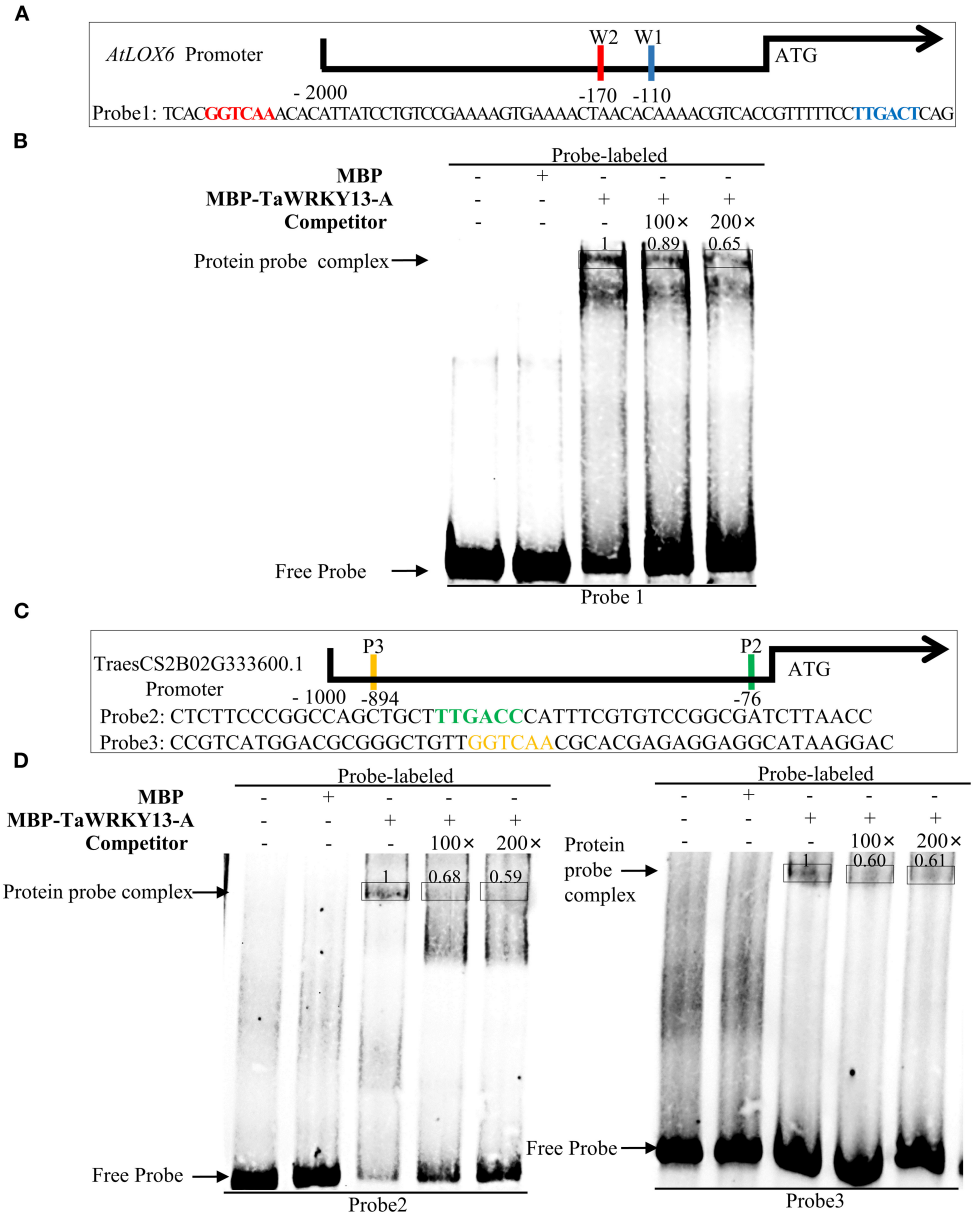
TaWRKY13-A can directly bind to the promoter region of *AtLOX6* and *TaLOX6*. **(A,C)** Positions of the W-box sites (with red, blue, yellow, and green colors) on promoters of *AtLOX6* and *TaLOX6* and probes (i.e., Probe1, Probe2, and Probe3) against W-box sites for EMSA. **(B,D)** Interactions of TaWRKY13-A protein and probes against *AtLOX6* and *TaLOX6* for EMSA experiment. The symbols of (+) and (–) indicated the presence and absence of specific probes, respectively. Numbers on the bands indicated the relative binding strength between MBP-TaWRKY13-A and labeled probes (The above experiments were repeated at least in three biological replicates).

As TaWRKY13-A affected the expression of some JA pathway genes, we further investigated whether TaWRKY13-A binds to the promoters of those genes. First, we scanned the 1-kb promoters of *AtLOX1, AtLOX2, AtLOX5*, and *AtLOX6* for the W-box motif (TTGACC/T), which is the main target site of WRKYs. We found that one and two W-box motifs lay in the promoter of *AtLOX1* and *AtLOX6*, respectively (Figure 7A, Supplementary Figure 9A). However, no W-box motif was identified in the *AtLOX5* promoter. Hence, we performed the EMSA to test the interaction between TaWRKY13-A and promoter of *AtLOX6* and *AtLOX1*. We fused the TaWRKY13-A to maltose-binding protein (MBP), and this MBP-TaWRKY13-A fusion as well as single MBP were expressed in *E. coli* (strain *Rosseta*). Then, we designed probe 1 (P1) against the promoter of *AtLOX6* and which specifically hybridizes with MBP-TaWRKY13-A but not MBP only, and this interaction could be completed by unlabeled probes (Figure 7B), whereas we found that the interaction between TaWRKY13-A and *AtLOX1* was not specific and competitive (Supplementary Figure 9B). This result suggested that TaWRKY13-A has the potential to bind *LOXs* and subsequently affects their expression.

**Figure 8:**
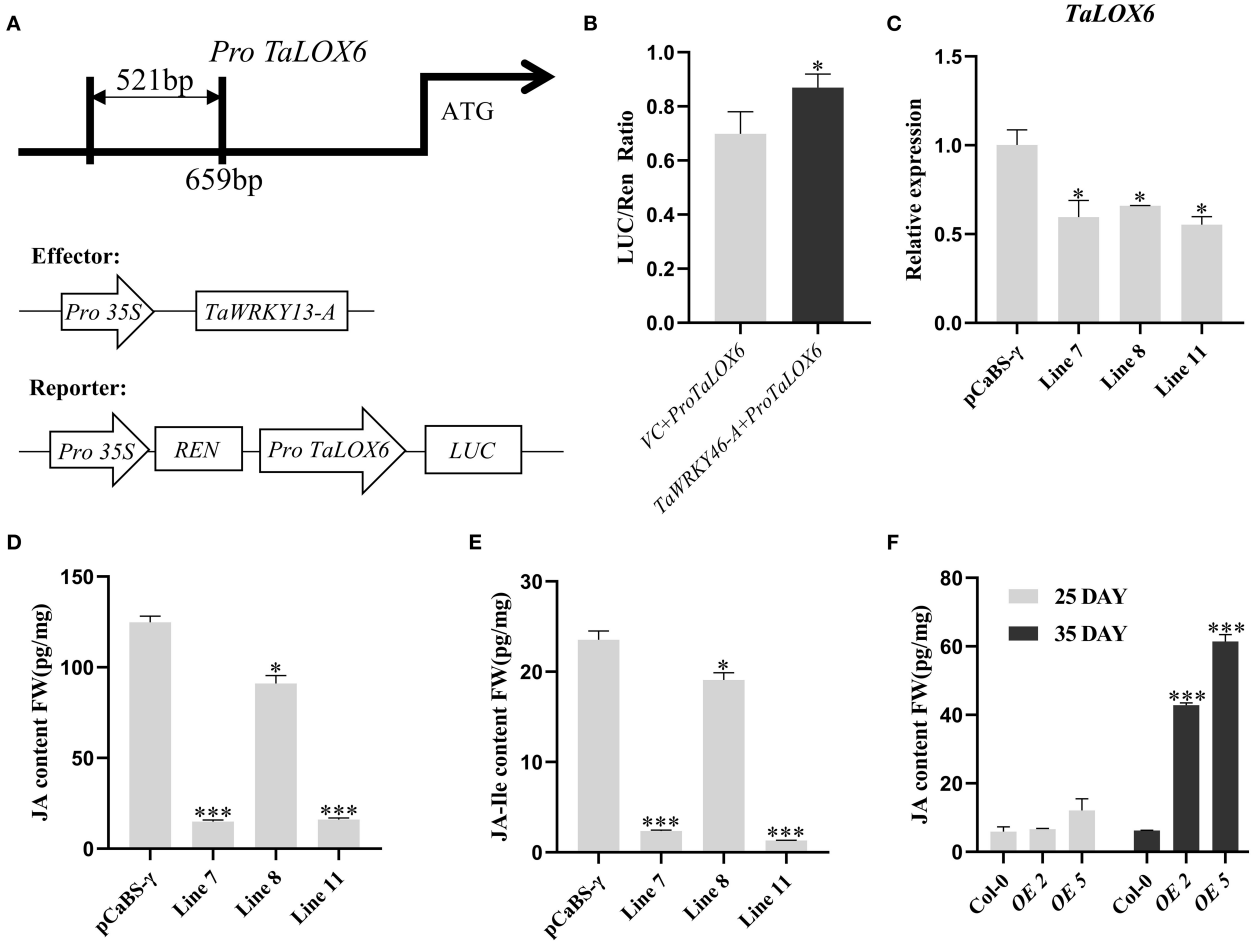
TaWRKY13-A can bind to the promoter region of *TaLOX6* in wheat protoplasts. **(A)** Diagram of fragment selected on *TaLOX6* and transient expression vectors. **(B)** The dual-luciferase reporter system shows that TaWRKY13-A can activate the *luciferase* gene linked to the *TaLOX6* promoter fragment, *Renilla* luciferase was used for normalization. The 35S:GFP acts as a negative control. **(C)** Expression of *TaLOX6* in *TaWRKY13*-*A*-silenced and VC wheat plants. **(D)** Measurement of endogenous JA content in 2-month-old *TaWRKY13*-*A*-silenced and VC wheat plants. **(E)** Measurement of endogenous JA-Ile content in 2-month-old *TaWRKY13*-*A*-silenced and VC wheat plants. **(F)** Measurement of endogenous JA content in 25-day-old and 35-day-old *TaWRKY13-A*-overexpressing *Arabidopsis*. (Error bars indicate SD. Asterisks indicate significant differences. Student's *t*-test, ^*^P < 0.05, ^***^P < 0.001. The above experiments were repeated at least in three biological replicates).

However, we aimed to clarify the mechanistic details of TaWRKY13-A-related leaf senescence in wheat. Thus, we searched the homologs of *AtLOX6* on WheatOmics (http://202.194.139.32/). We performed the sequence blast with the Pfam (PF00305) number of lipoxygenase family and conducted a phylogenetic analysis based on our RNA-seq data at four developmental stages (i.e., YL, ML, ES, and LS) of wheat leaf (Supplementary Figure 8A). We found a gene (TraesCS2B02G333600.1) showing the highest similarity with *AtLOX6* and hence named *TaLOX6*, which is increasing during leaf senescence and has not been functionally characterized before (Supplementary Figure 8B). We identified two W-box elements in the promoter region of *TaLOX6* (Figure 7C). Thus, we planned to analyze the interaction between TaWRKY13-A and *TaLOX6* by using the EMSA and luciferase reporter system. First, we synthesized probe 2 (P2) and probe 3 (P3) both harboring one W-box motif on the promoter of *TaLOX6* (Figure 7C). Both P2 and P3 showed the specific and competitive interaction with MBP-TaWRKY13-A protein but not MBP only (Figure 7D). Furthermore, we confirmed the interaction between MBP-TaWRKY13-A and *TaLOX6* by using the luciferase reporter system. *LUC* gene driven by the promoter of *TaLOX6* was co-transformed with *35S:TaWRKY13-A-GFP* into wheat protoplasts (Figure 8A). The reaction catalyzed by LUC was quantification, indicating the bond strength between TaWRKY13-A and *TaLOX6* promoter. TaWRKY13-A-GFP but not GFP alone was able to elevate the ratio of LUC activity to REN activity (internal reference) (Figure 8B). These data further manifested TaWRKY13-A can bind to *LOXs* in wheat. Meanwhile, we also proved that the expression of *TaLOX6* was suppressed in *TaWRKY13-A*-silenced wheat plants compared with VC plants (Figure 8C). This result suggested that TaWRKY13-A is the potential to regulate *TaLOX6 in vivo*.

Less was known about the functional role of *TaLOX6*. In this study, we preliminarily analyzed the responses of *TaLOX6* to MeJA treatment. We verified that the expression level of *TaLOX6* was induced by MeJA treatment (Supplementary Figure 8C). Despite this, more studies are needed to verify that TaLOX6 is involved in the regulation of leaf senescence by cooperating with TaWRKY13-A. To further validate TaWRKY13-A regulating leaf senescence *via* the JA pathway, we measured the content of JA and JA-Ile between *TaWRKY13-A*-silenced and VC wheat plants in Supplementary Figure 4 by using the LC-MS/MS method. In line with the phenotypic differences among the above plants after dark treatment, we detected the significantly lower levels of JA and JA-Ile in *TaWRKY13-A*-silenced wheat plants than that in VC plants (Figures 8D,E). Meanwhile, we also measured the JA content in *TaWRKY13-A*-overexpressing *Arabidopsis* plants and Col-0 at juvenile and senescent stages (Figure 8F). Notably, we detected a slightly higher JA level in *TaWRKY13-A*-overexpressing young leaves than Col-0, while the JA level elevated in senescent leaves of *TaWRKY13-A*-overexpressing plants more dramatically than that in Col-0 (Figure 8F). This result demonstrated that leaf senescence onset promoted by TaWRKY13-A involves the activation of JA biosynthesis. Nevertheless, relevant experimental studies in wheat will be much more helpful for assessing the regulatory network of TaWRKY13-A in future.

### TaWRKY13-A Is Possibly Partially Functional Conserved With AtWRKY53

**Figure 9:**
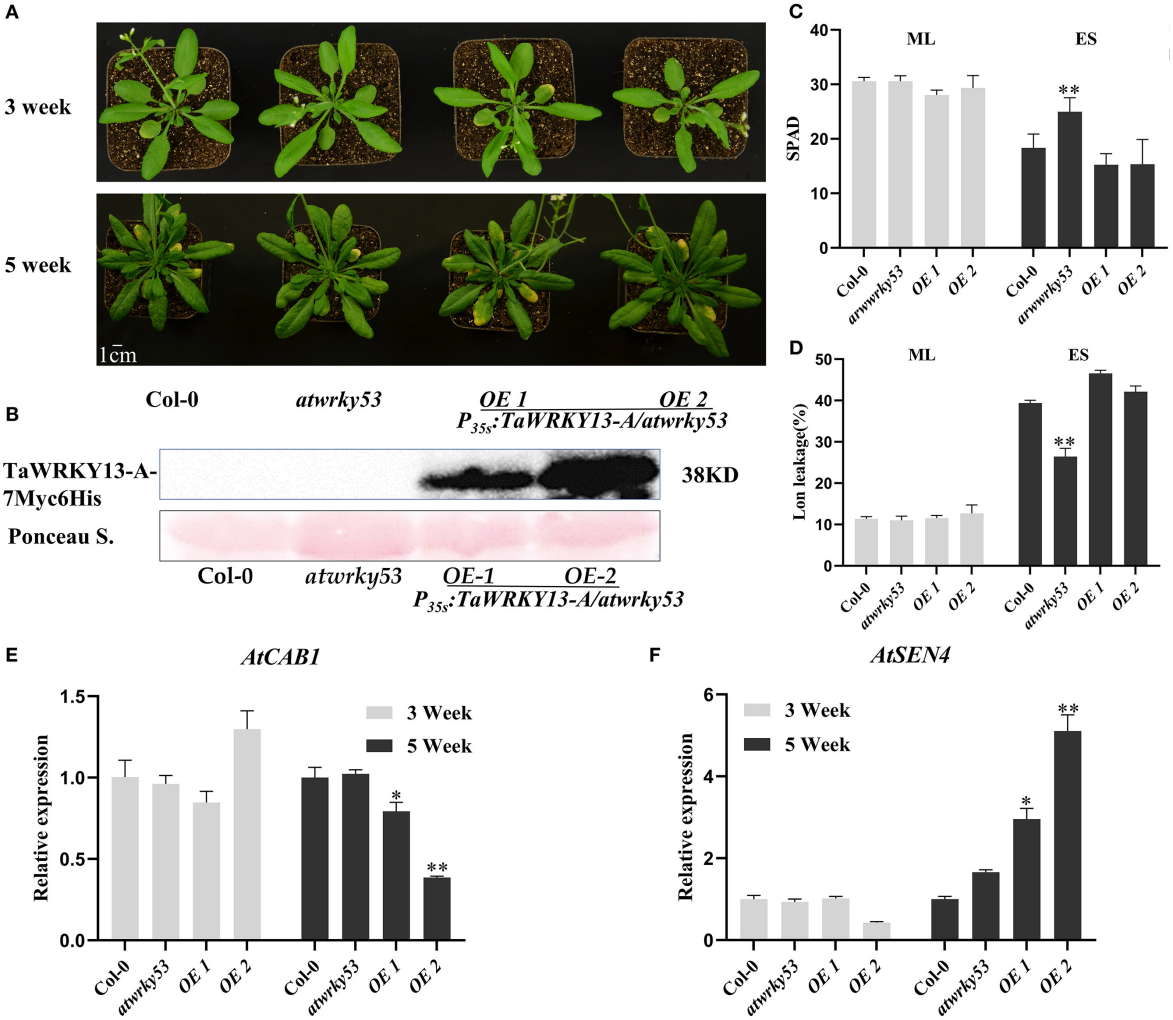
Overexpression of *TaWRKY13-A* rescues delayed leaf senescence in *atwrky53* mutant. **(A)** Phenotypic observation of *P*_35*s*_*:TaWRKY13-A-7Myc6His*/*atwrky53*, Col-0, and *atwrky53 Arabidopsis* plants. **(B)** Identification of TaWRKY13-A-7Myc6His in *P*_35*s*_*:TaWRKY13-A-7Myc6His*/*atwrky53*, Col-0, and *atwrky53 Arabidopsis* by using the Western blot. **(C,D)** Measurement of chlorophyll content **(C)** and ion leakage rate **(D)** in **(A)**. **(E,F)** Detection of senescence-associated genes including an upregulated gene *AtSEN4* **(F)** and a downregulated gene *AtCAB1* **(E)** in the above plants. (Error bars indicate SD. Asterisks indicate significant differences. Student's *t*-test, ^*^P < 0.05, ^**^P < 0.01, and *n* ≥ 30. The above experiments were repeated at least in three biological replicates).

Based on the phylogenetic analysis among those published senescence-related WRKYs, we identified that TaWRKY13-A shares a relatively high similarity with AtWRKY53, and they both belong to the group III WRKYs (Supplementary Figure 1A). Besides, many studies have revealed the crucial role of AtWRKY53 in the regulation of leaf senescence and the connection between AtWRKY53 and JA pathway (Miao and Zentgraf, 2007). Thus, we investigated the functional conservation between TaWRKY13-A and AtWRKY53. Subsequently, we generated a construct where the CDS of TaWRKY13-A was driven by CaMV 35S promoter, and it was introduced into the *atwrky53* mutant. After confirming the expression of TaWRKY13-A-7Myc6His by Western blot, the process of leaf senescence was compared among Col-0, *atwrky53* mutant, and *atwrky53* mutant harboring 35S:*TaWRKY13-A-7Myc6His* construct (Figure 9B). Three-week-old above plants showed parallel growth phenotypes, while after growing for 5 weeks, *atwrky53* exhibited a delayed leaf senescence phenotype compared with Col-0 and *35S:TaWRKY13-A-7Myc6His*/*atwrky53* (Figure 9A). Chlorophyll content, ion leakage rate, and expression of two senescence-related genes, namely, *AtCAB1* and *AtSEN4*, were detected before and after leaf senescence onset, which were in line with the senescence-related phenotypic alterations among the above plants (Figures 9C–F). This result suggested that TaWRKY13-A may be partially conserved with AtWRKY53 under the natural growth condition.

## Discussion

Although numerous studies have identified multidimensional regulators of senescence, the regulatory network of lifespan remains a mystery. Plant senescence is obviously distinguished from the aging in animals, which is mainly reflected in the decline of assimilation and the nutrients redistribution. Moreover, the telomere length has been recently demonstrated to be positively related to early flowering time, while longer telomere in animals usually means longevity (Choi et al., 2021). Therefore, appropriate timing of leaf senescence is of immense importance for plant growth and especially for crop yield and quality.

To date, researchers have addressed the roles of various genes related to leaf senescence in phytohormone pathways, transcriptional regulation, epigenetic modification, autophagy, circadian clock, DNA damage repair, and chlorophyll metabolism (Woo et al., 2019). Among these regulators of leaf senescence, TFs are one of the most intensively studied gene families (Schippers, 2015). TFs function as a key step for integrating senescence-related signals and further executing the precise transcriptional modulation of diverse senescence-related genes. WRKYs are one of the largest TF families in higher plants and play important roles mainly in the responses to biotic and abiotic stresses, carbohydrate synthesis, leaf senescence, development, and secondary metabolism (Rushton et al., 2010; Viana et al., 2018). Some studies have revealed the connection between WRKYs and phytohormones in the regulation of leaf senescence. Moreover, some experimental data suggested that there is a feedback loop among leaf senescence, phytohormones, and WRKYs, which makes the regulation of WRKYs in leaf senescence more sophisticate (Miao and Zentgraf, 2007; Jiang et al., 2014; Guo et al., 2017; Kim et al., 2019; Zhao et al., 2020b). This fine-tuning also suggests that the progression of leaf senescence is a highly ordered process. In this study, TaWRKY13-A promotes the accumulation of JA mainly by activating the transcription of *LOXs*. Notably, the transcription of *TaWRKY13-A* is also activated by MeJA treatment and leaf senescence onset, which implies that *TaWRKY13-A* may be under the control of a feedback loop. Nevertheless, more data are needed for verifying this hypothesis.

WRKYs show their multiple roles in various biological events, which partially results from their potentials to interact with numerous target genes. Meanwhile, the single WRKY protein could also bind to diverse promoters to mediate different signals. In this study, we focused on the regulation of TaWRKY13-A on the expression of *LOXs* during leaf senescence. It has been known that *LOXs* encode lipoxygenases and participate in JA biosynthesis (Wasternack and Song, 2016). In *Arabidopsis, LOX2* is responsible for JA biosynthesis and under the regulation of TCP4 in JA-induced leaf senescence, which is simultaneously repressed by miRNA319 (Schommer et al., 2008; Koyama et al., 2017). Our data showed that TaWRKY13-A prefers *AtLOX6* and its ortholog in wheat as target genes during leaf senescence. Previously, AtLOX6 has been reported to function in the responses to long-distance wound signaling and stress resistance (Chauvin et al., 2013; Grebner et al., 2013). However, *TaLOX6* has never been functionally characterized in wheat. In this study, we proved that the expression of *TaLOX6* is affected by MeJA treatment and senescence process, which suggests that *TaLOX6* is possibly related to JA-induced leaf senescence, whereas the overexpression and impairment of *TaLOX6* in wheat will be extremely helpful for identifying the function of *TaLOX6* in future.

The JA pathway has long been acknowledged for its role in leaf senescence onset. However, some studies also reveal the complexity of JA-related senescence. For instance, mutants of some JA signaling components, such as *coi1-1*, show indistinguishable senescence phenotypes compared with WT *Arabidopsis* plants (He et al., 2002a; Seltmann et al., 2010). In this study, we predominately focused on the connection between TaWRKY13-A and JA biosynthesis. Notably, the expression level of some JA signaling components, including *MYC2, MYC3*, and *MYC4*, were altered in *TaWRKY13-A*-overexpressing *Arabidopsis* plants. To further assess whether this affection is directly carried out by TaWRKY13-A, the analysis of the interaction between TaWRKY13-A and *MYCs* in *Arabidopsis* and wheat is essential. Moreover, since *TaWRKY13-A* itself is regulated by MeJA, the potential of TaWRKY13-A as a target gene of JA-related TFs is also considerable.

Studies on functional genes in wheat are lagging behind those in other crops, mainly due to their allohexaploid genome and the high similarity among the paralogs. By using the BSMV–VIGS method, we planned to specifically silence the transcription of *TaWRKY13-A*. In fact, there is just a single nucleotide difference in CDS between TaWRKY13-A and TaWRKY13-D and which even does not alter the amino acid sequence. Thus, we conducted the phenotypic analysis to investigate the role of TaWRKY13-A by choosing the wheat plants with significantly decreased *TaWRKY13-A* and irregularly changed *TaWRKY13-B* and *TaWRKY13-D* among those infected seedlings. It is conceivable that the delayed leaf senescence phenotype in VIGS plants may be an eventual outcome caused by decreased *TaWRKY13s*. More importantly, although the overexpression of *TaWRKY13-A* led precious leaf senescence in *B. distachyon* and *Arabidopsis*, it is hard to demonstrate whether TaWRKY13-A and TaWRKY13-B are completely redundant. Moreover, as there is an extremely high similarity between *TaWRKY13-A* and *TaWRKY13-B*, specific studies on each gene by CRSIPR-Cas technology are difficult. Even so, we found that the upstream regulatory sequences of *TaWRKY13-A* and *TaWRKY13-B* are distinct, and their expression profiles and abundance of transcripts during leaf senescence are also very different (Supplementary Figure 2). These data implied that TaWRKY13-A and TaWRKY13-B may play diverse roles in wheat. However, details about how TaWRKY13-B and TaWRKY13-D participating in the regulation of leaf senescence will help us to comprehensively understand the roles of TaWRKY13s in wheat leaf senescence and whether they are functionally redundant.

To data, WRKYs are divided into seven groups based on the number of WRKY domains and the type of zinc finger structures. According to this classification, TaWRKY13-A belongs to group III WRKYs, which also contains a key regulator of leaf senescence, AtWRKY53. Our results indicated that TaWRKY13-A has the potential to rescue the delayed leaf senescence phenotype in *atwrky53* mutants. These data suggested that TaWRKY13-A is partially functional conserved with AtWRKY53 in age-dependent leaf senescence. Previously, AtWRKY53 has been demonstrated to relate with the JA pathway by interacting with a JA-inducible protein ESR/ESP in leaf senescence (Miao and Zentgraf, 2007). Here, we concluded that TaWRKY13-A also regulates leaf senescence by modulating JA biosynthesis, whereas whether TaWRKY13-A regulates leaf senescence in a comparable way with AtWRKY53 is needed to be determined in future. To data, as few regulators of leaf senescence have been characterized in wheat, whether the underlying mechanisms of phytohormones-related leaf senescence are similar to those in other model plants, such as *Arabidopsis* and rice, remain to be proved (Sultana et al., 2021). Collectively, we identified a novel activator of wheat leaf senescence, TaWRKY13-A, which accelerates leaf senescence by promoting JA biosynthesis, and is partially functional conserved with AtWRKY53 in age-dependent leaf senescence. Moreover, TaWRKY13-A could be a new clue for molecular breeding in wheat.

## Data Availability Statement

The datasets presented in this study can be found in online repositories. The names of the repository/repositories and accession number(s) can be found in the article/Supplementary Material.

## Author Contributions

CZ and GW conceived and designed the experiments. HQ, LC, and XG generated the constructs and transgenic lines. LC, YL, and SZ tested the transcriptional activity and performed the phenotypic and physiological analyses. KL conducted the bioinformatics analysis. HQ performed the EMSA experiment. HQ, PY, CZ, and GW wrote the manuscript. All authors read and approved the final manuscript.

## Conflict of Interest

The authors declare that the research was conducted in the absence of any commercial or financial relationships that could be construed as a potential conflict of interest.

## Publisher's Note

All claims expressed in this article are solely those of the authors and do not necessarily represent those of their affiliated organizations, or those of the publisher, the editors and the reviewers. Any product that may be evaluated in this article, or claim that may be made by its manufacturer, is not guaranteed or endorsed by the publisher.
