# Supplementary figures and images for: TaWRKY13-A Serves as a Mediator of Jasmonic Acid-Related Leaf Senescence by Modulating Jasmonic Acid Biosynthesis
Source: Front Plant Sci. 2021 Sep 1;12:717233. doi: 10.3389/fpls.2021.717233 (PMC8442999; doi:10.3389/fpls.2021.717233)

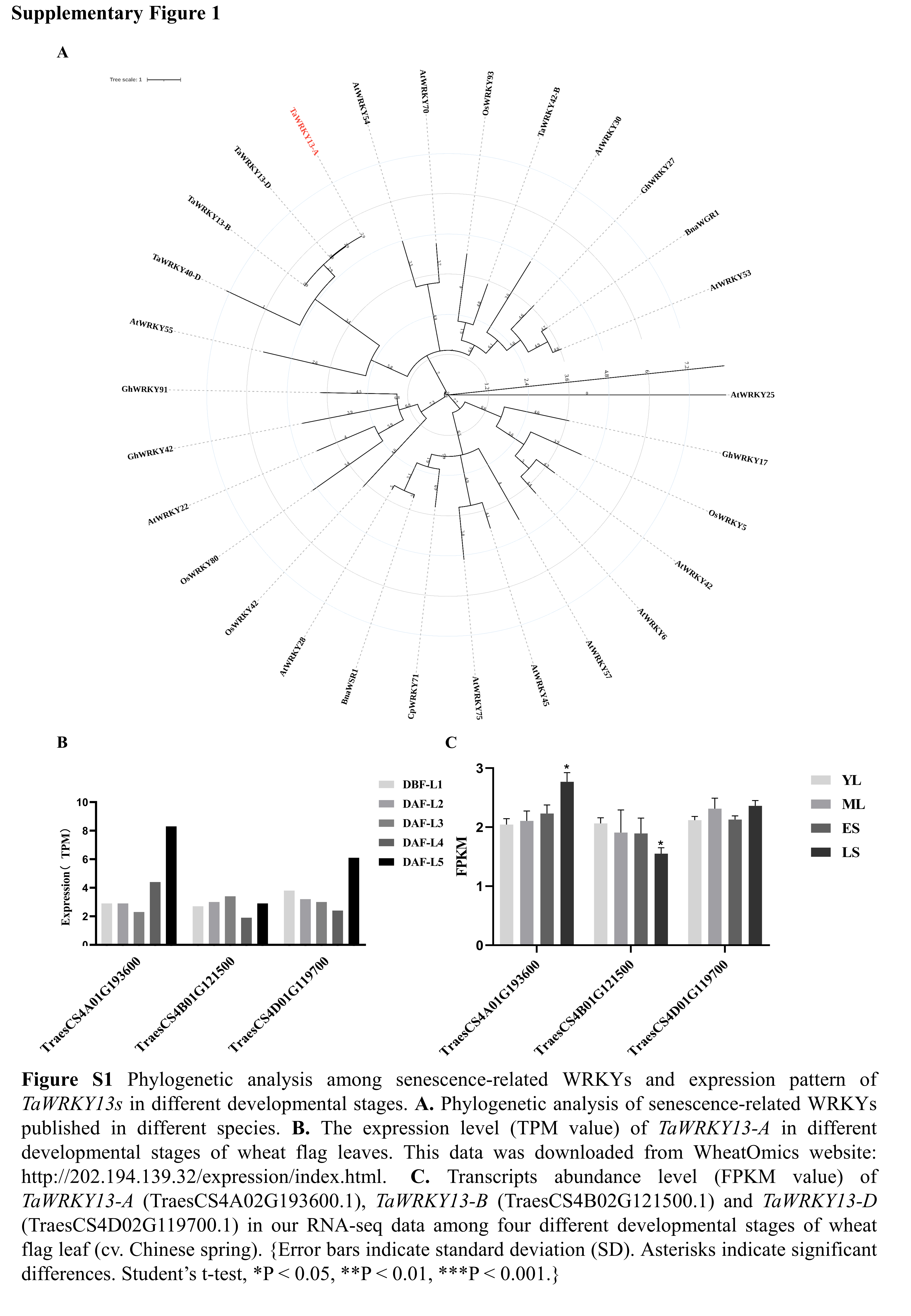

Supplement: Supplementary file 1 [file Image_1.TIF]

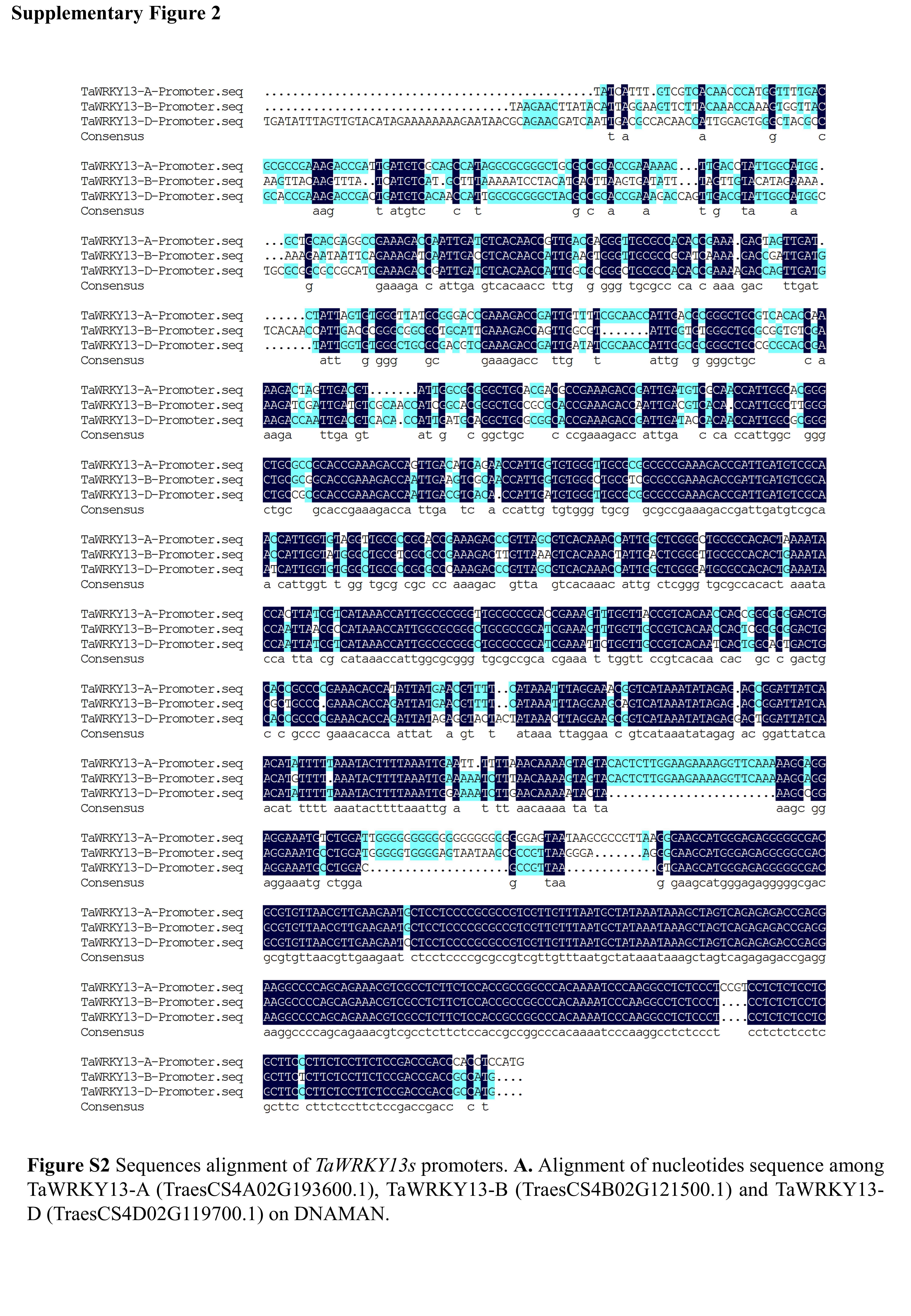

Supplement: Supplementary file 2 [file Image_2.TIF]

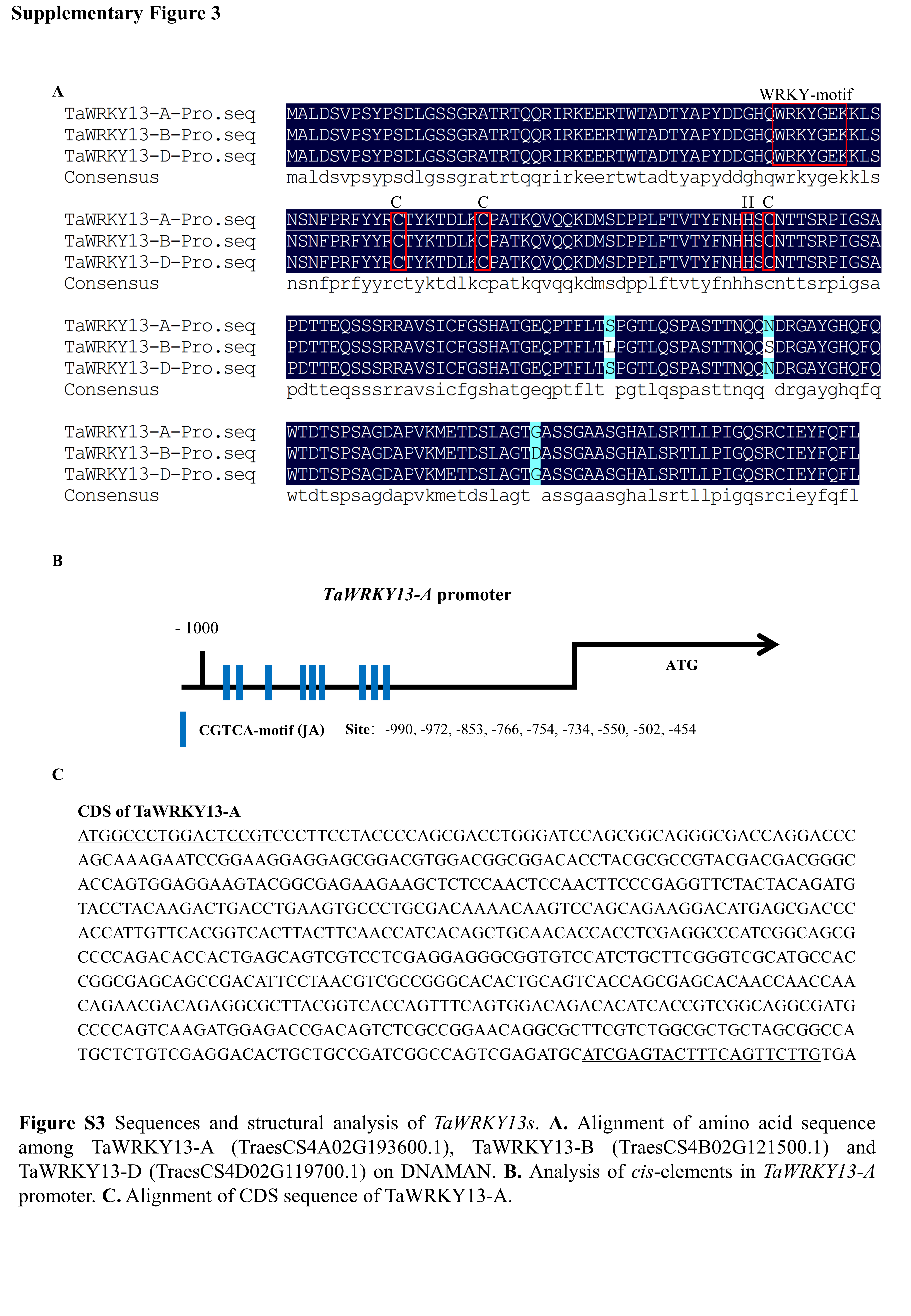

Supplement: Supplementary file 3 [file Image_3.tif]

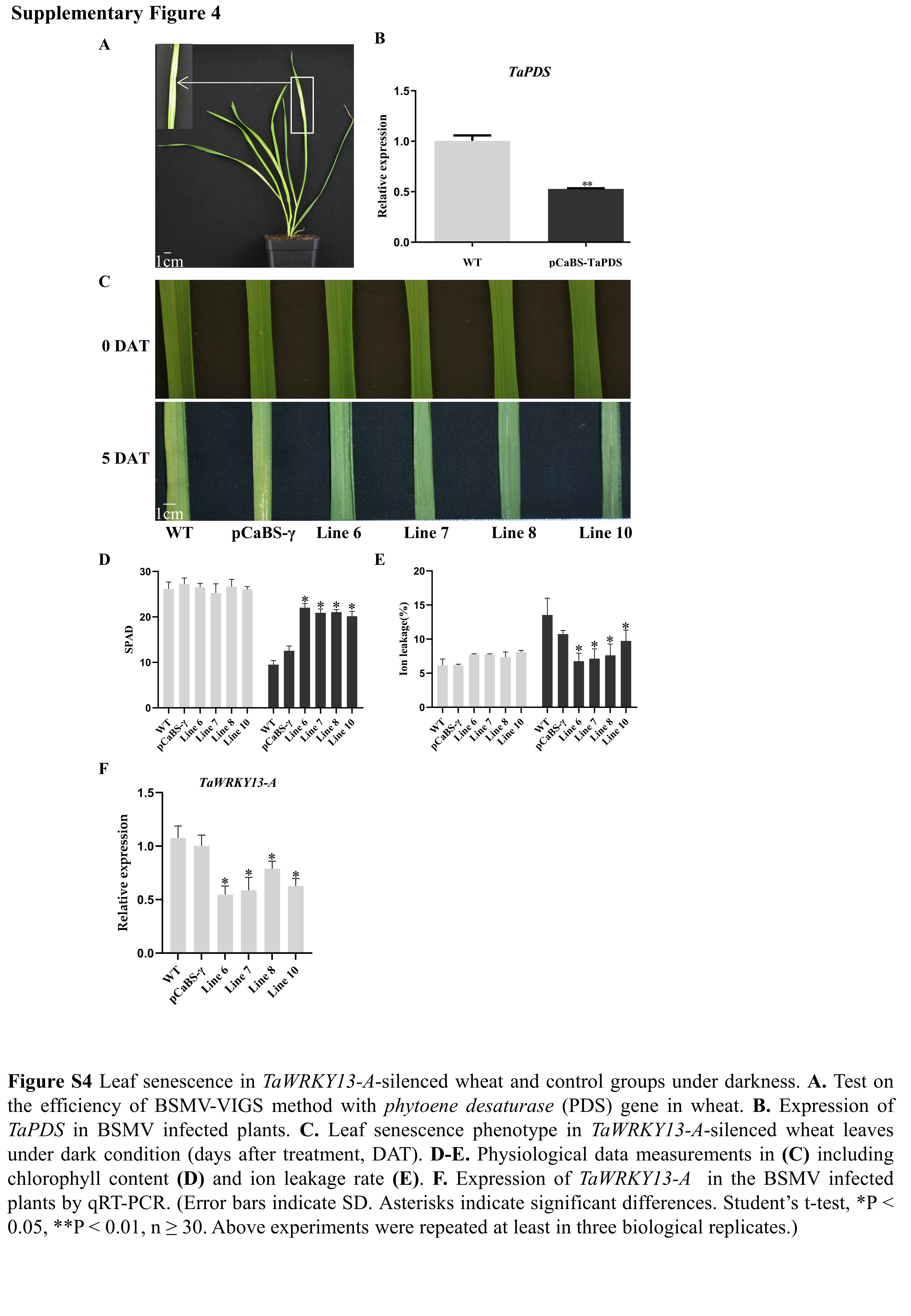

Supplement: Supplementary file 4 [file Image_4.tif]

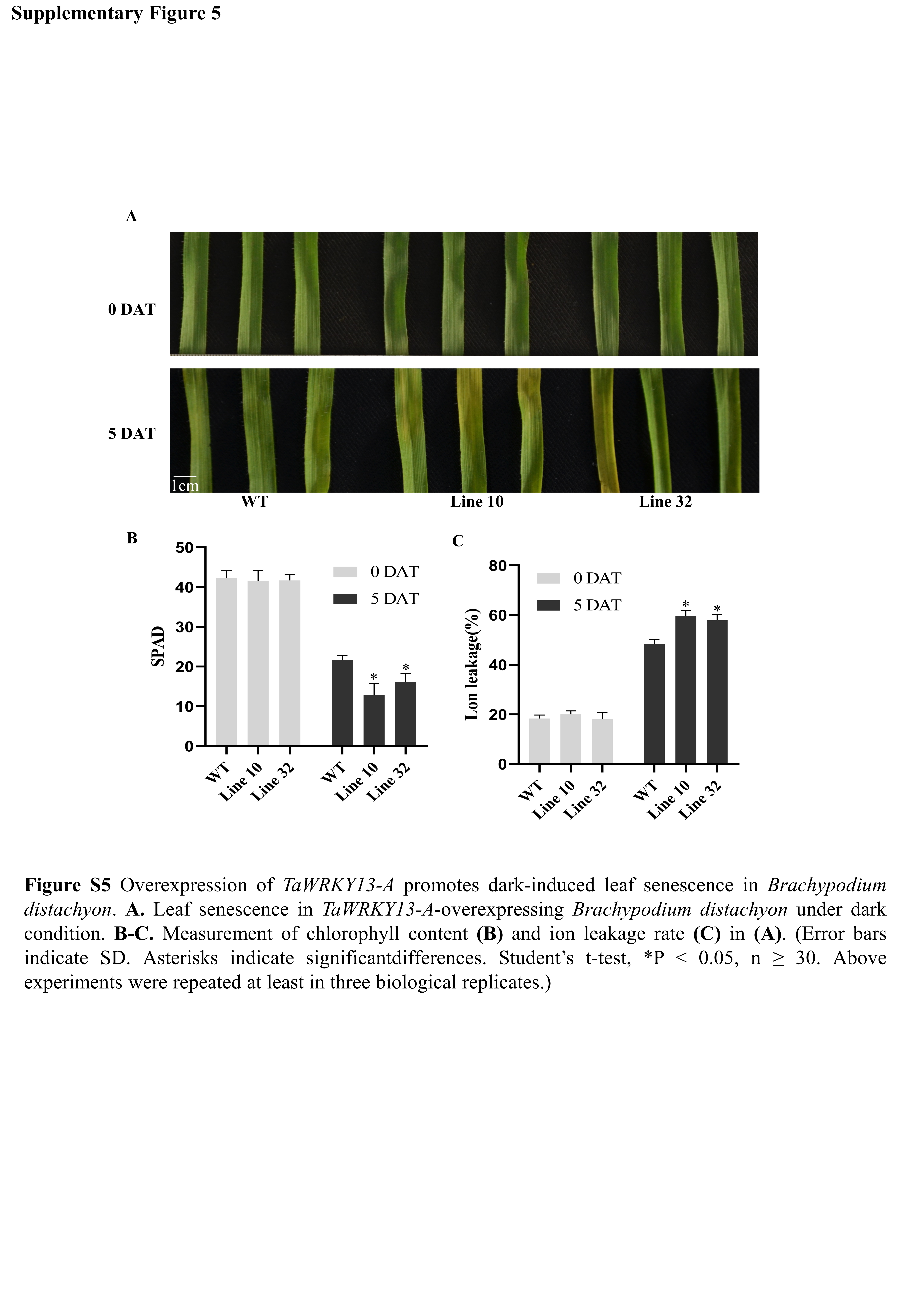

Supplement: Supplementary file 5 [file Image_5.tif]

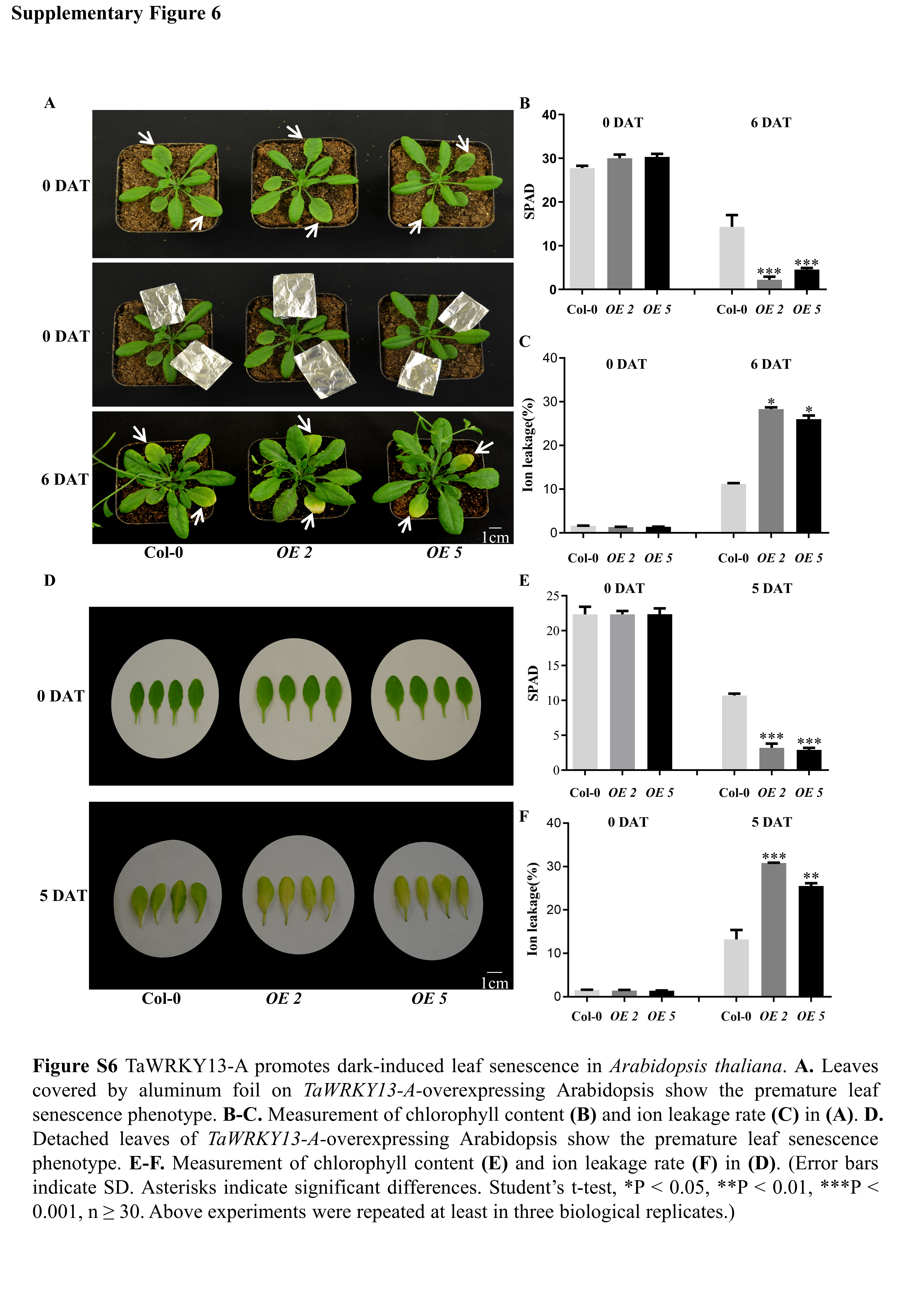

Supplement: Supplementary file 6 [file Image_6.TIF]

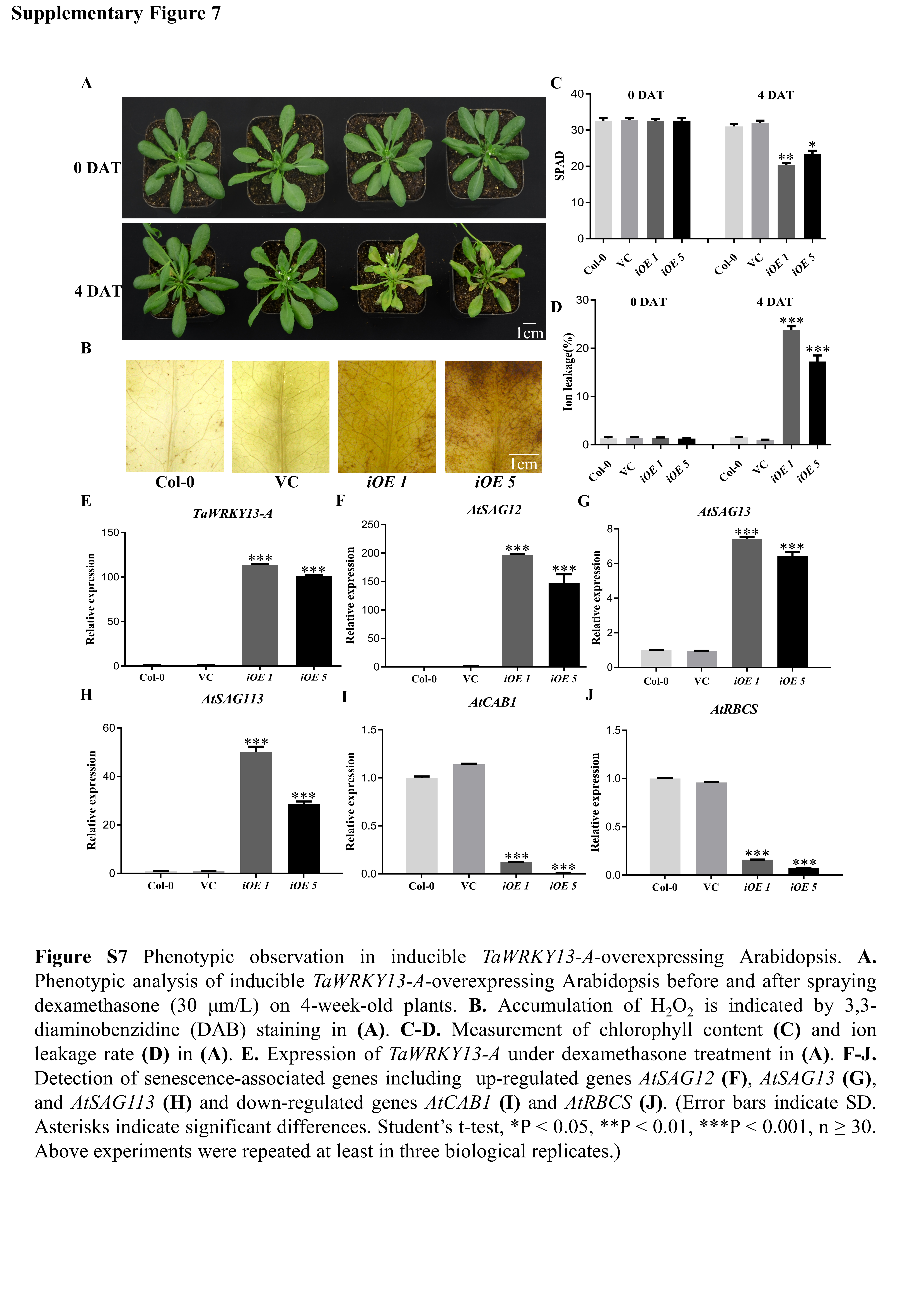

Supplement: Supplementary file 7 [file Image_7.TIF]

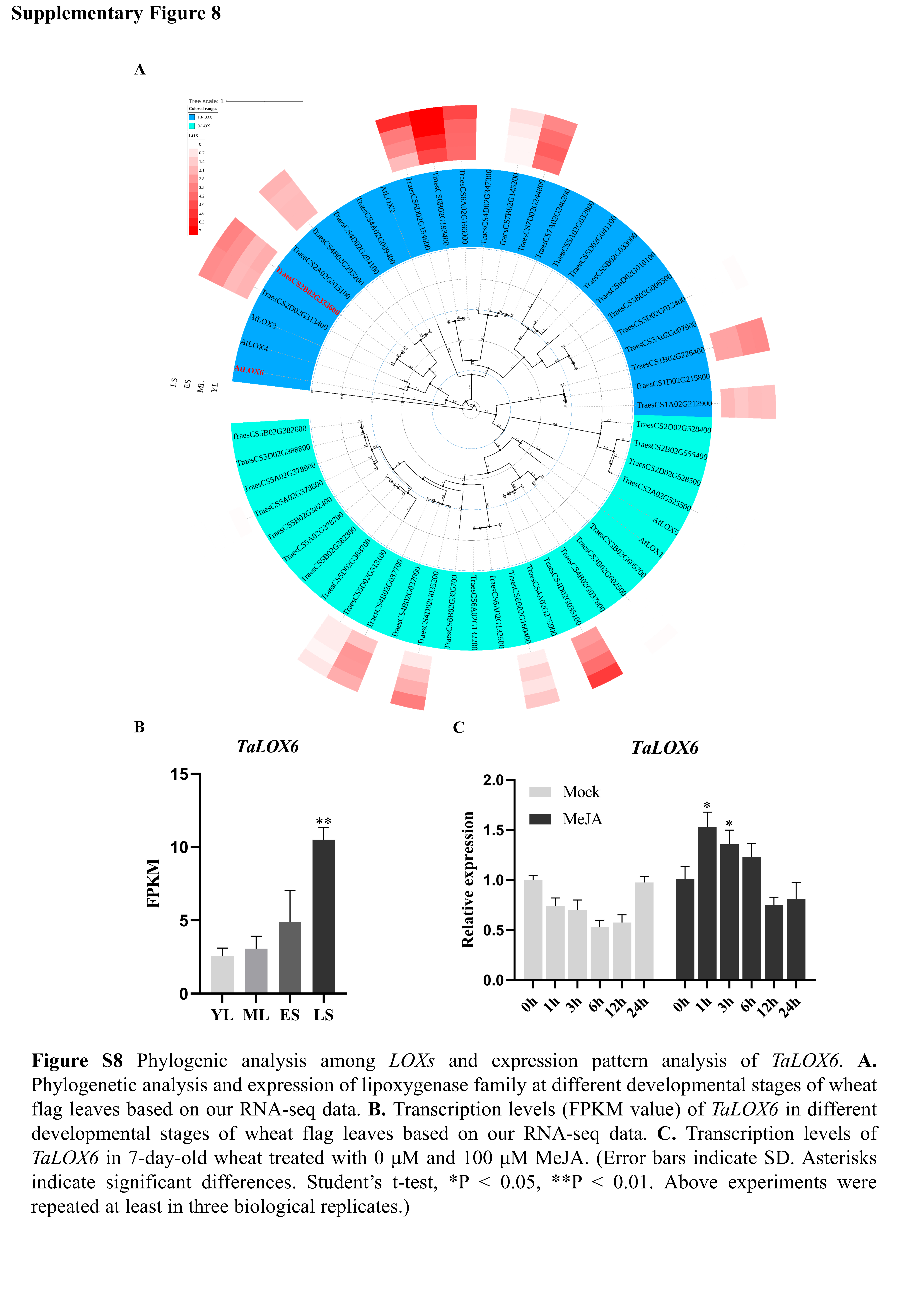

Supplement: Supplementary file 8 [file Image_8.tif]

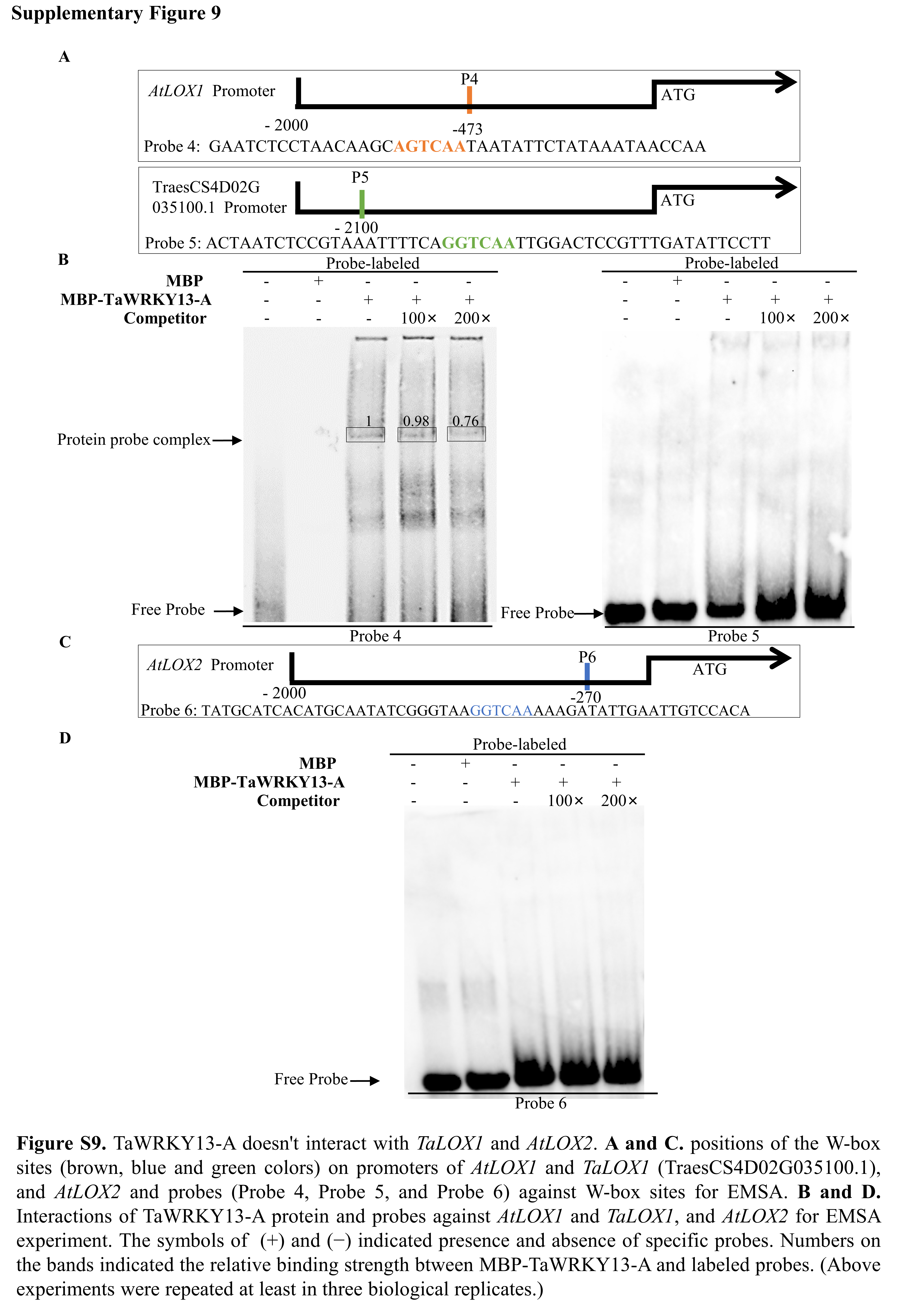

Supplement: Supplementary file 9 [file Image_9.TIF]

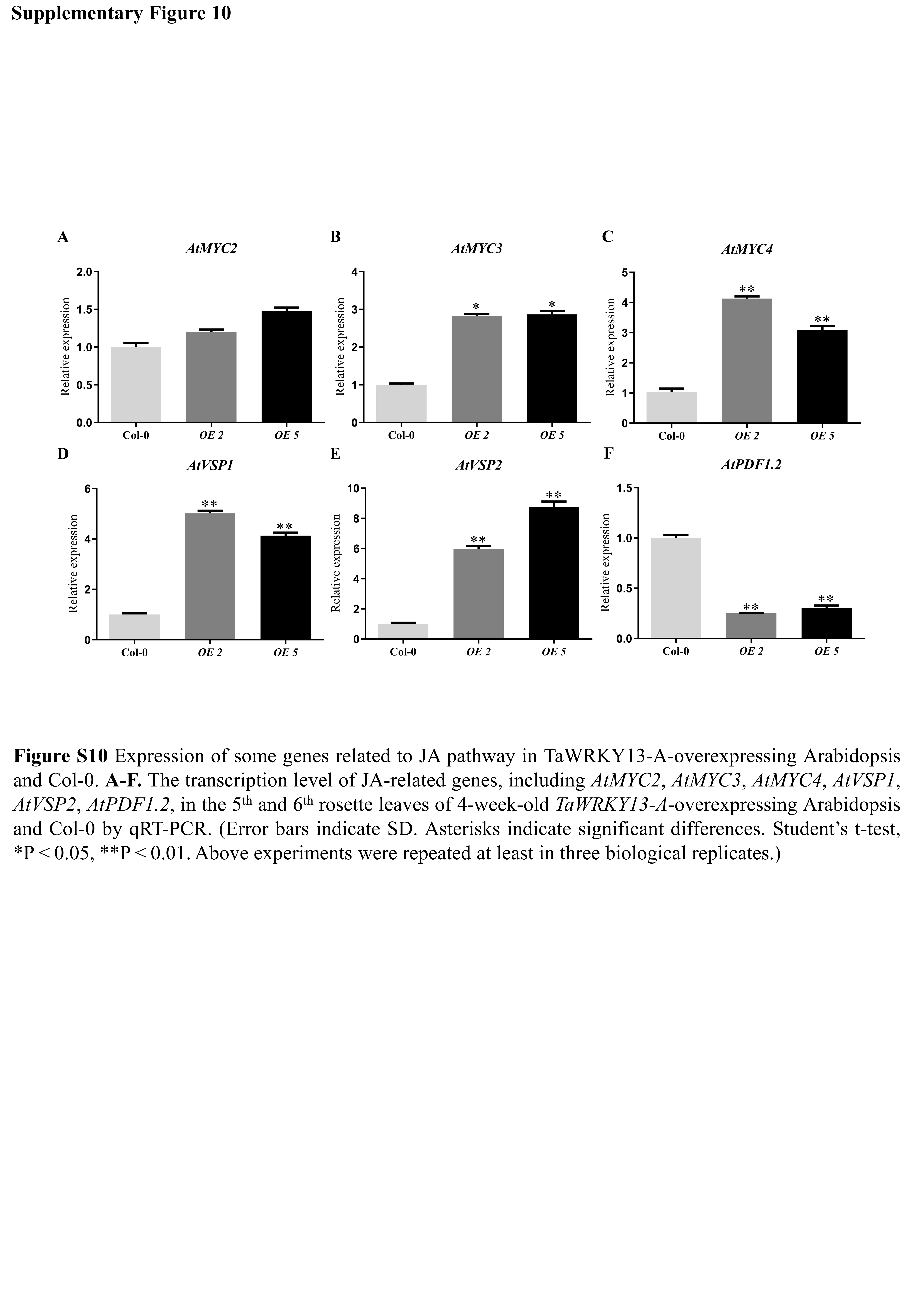

Supplement: Supplementary file 10 [file Image_10.tif]
